# Silicon‐ and Germanium‐Functionalized Perylene Diimides: Synthesis, Optoelectronic Properties, and Their Application as Non‐fullerene Acceptors in Organic Solar Cells

**DOI:** 10.1002/chem.202301337

**Published:** 2023-08-28

**Authors:** Bettina Schlemmer, Aileen Sauermoser, Sarah Holler, Elena Zuccalà, Birgit Ehmann, Matiss Reinfelds, Roland C. Fischer, Heinz Amenitsch, Jose M. Marin‐Beloqui, Lucie Ludvíková, Tomáš Slanina, Michael Haas, Thomas Rath, Gregor Trimmel

**Affiliations:** ^1^ Institute for Chemistry and Technology of Materials, NAWI Graz Graz University of Technology Stremayrgasse 9 8010 Graz Austria; ^2^ Institute of Inorganic Chemistry, NAWI Graz Graz University of Technology Stremayrgasse 9 8010 Graz Austria; ^3^ Department of Physical Chemistry University of Málaga Blvrd Louis Pasteur 31 29010 Málaga Spain; ^4^ Institute of Organic Chemistry and Biochemistry of the Czech Academy of Sciences Flemingovo nám. 2 16610 Prague 6 Czech Republic

**Keywords:** donor-acceptor systems, group 14 elements, organic photovoltaics, organometallics, perylene diimides

## Abstract

Organic solar cells have been continuously studied and developed through the last decades. A major step in their development was the introduction of fused‐ring non‐fullerene electron acceptors. Yet, beside their high efficiency, they suffer from complex synthesis and stability issues. Perylene‐based non‐fullerene acceptors, in contrast, can be prepared in only a few steps and display good photochemical and thermal stability. Herein, we introduce four monomeric perylene diimide acceptors obtained in a three‐step synthesis. In these molecules, the semimetals silicon and germanium were added in the bay position, on one or both sides of the molecules, resulting in asymmetric and symmetric compounds with a red‐shifted absorption compared to unsubstituted perylene diimide. Introducing two germanium atoms improved the crystallinity and charge carrier mobility in the blend with the conjugated polymer PM6. In addition, charge carrier separation is significantly influenced by the high crystallinity of this blend, as shown by transient absorption spectroscopy. As a result, the solar cells reached a power conversion efficiency of 5.38 %, which is one of the highest efficiencies of monomeric perylene diimide‐based solar cells recorded to date.

## Graphical abstract

**In with the old**: Perylene diimide, one of the oldest electron acceptors, was functionalized with group 14 elements in the bay position. The influence of these substitutions on their properties and performance in organic photovoltaics was thoroughly studied.

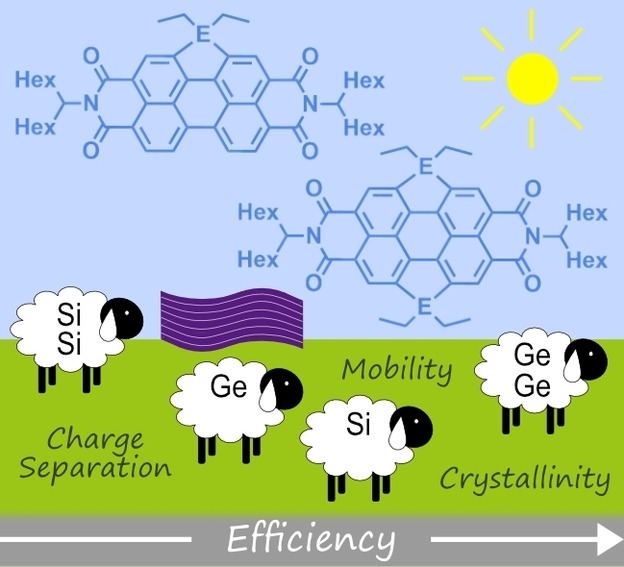

## Introduction

Due to climate change, the demand for energy from renewable resources is continually rising. Among the renewable resources, the sun is one of the most promising for energy production, considering the high power of the solar irradiance and its large geographical availability.^[1]^ Currently, the most widely applied solar cell technique is based on crystalline silicon.^[2]^ However, emerging solar technologies have become more efficient throughout the last years,[^3^, ^4^] due to the extensive research in this field. A highly promising emerging technology is organic photovoltaics (OPVs) based on organic molecules assembled in thin, lightweight, and sometimes flexible and semitransparent solar cell devices.[^5^, ^6^]

Organic solar cells (OSCs) are already approaching 20 % power conversion efficiency (PCE),[^4^, ^7^] which is largely based on the development of non‐fullerene acceptors, especially fused ring electron acceptors. But these molecules are difficult to synthesize, leading to high production costs and long preparation times. In addition, the device stability remains one of the drawbacks of OSCs, requiring further research.^[8]^ Therefore, researchers have been investigating simpler molecules with a lower synthetic complexity ranging from similar unfused structures,^[9]^ through porphyrins^[10]^ to perylenes,[^6^, ^11^, ^12^, ^13^, ^14^] to name a few.

Perylene based non‐fullerene acceptors were applied in OSCs due to their favorable optical and electrochemical properties, such as a high absorption in the visible range (*ϵ* between 10^4^–10^6^ M^−1^ cm^−1^),^[15]^ high temperature and photo‐stability, and the easy modifiable structure. Tang used a perylene derivative in one of the first OSC devices.^[16]^ Since then, extensive research was performed on perylene imides for several applications in solar cells, for example, as acceptor material or as cathode interlayer in OSCs,[^6^, ^17^] sensitizers in dye sensitized solar cells (DSSCs)^[18]^ or as cathode interlayer in perovskite solar cells.^[19]^ In OSCs, starting from simple modifications on the imide, *peri*, *ortho* and bay positions, the trend went to linking two or more perylene units to obtain a larger conjugated system.[^11^, ^13^, ^20^] This strategy of using a larger conjugated system or more than one chromophoric center increased the efficiency, yielding PCEs for perylene diimide‐based organic solar cells of up to 12.6 %.^[21]^ However, the increase in acceptor size did not only improve the efficiency, but also raised the overall synthetic complexity and accordingly their costs. As a result, they become less applicable in large scale. In this context, simpler perylene structures derived through easier synthesis with fewer steps would be beneficial. Several monomeric perylene diimide acceptors have been investigated as acceptors in organic solar cells, but so far only approximately 10 % of them reached PCE values above 5 %.[^14^, ^22^]

Herein, we introduce four novel electron acceptors based on monomeric perylene diimides with the semimetals silicon and germanium fused to the backbone in the bay position. These structures were derived through an easy to perform and fast three‐step synthesis. The materials were optically and electrochemically characterized before they were used to fabricate solar cells. The photovoltaic devices and the absorber layers were then characterized using current voltage curves, light intensity dependent measurements of photocurrent and photovoltage, atomic force microscopy, grazing incidence wide angle X‐ray scattering (GIWAXS), as well as femtosecond transient absorption spectroscopy (fs‐TAS).

## Results and Discussion

### Synthesis

**Figure 1:**
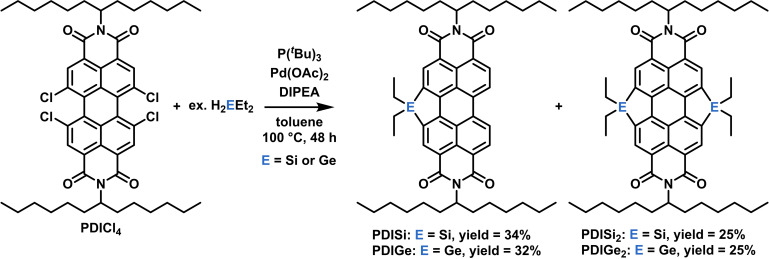
Synthesis scheme for the reaction of PDICl_4_ to the respective acceptors PDISi, PDIGe, PDISi_2_ and PDIGe_2_.

**Figure 2:**
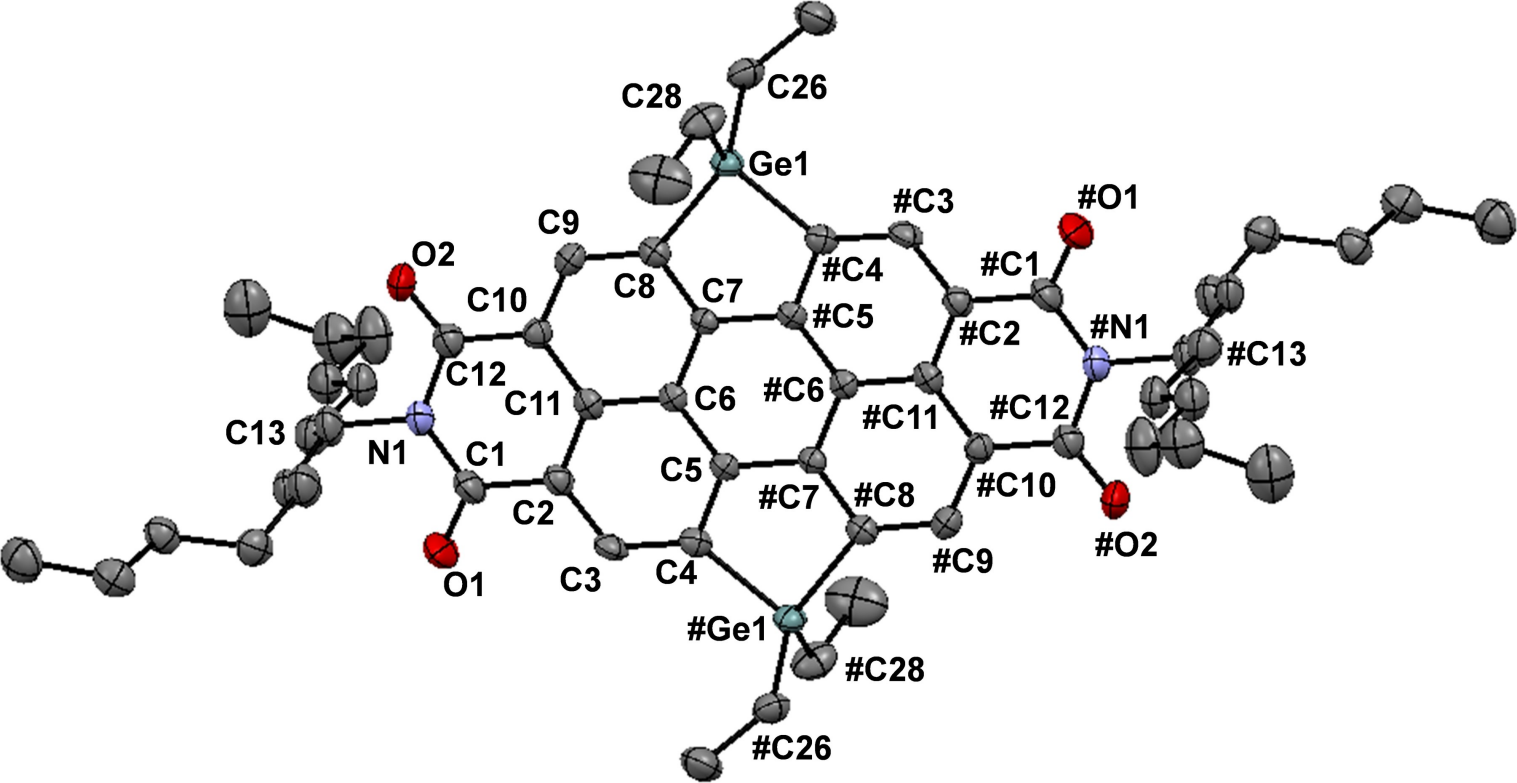
ORTEP for PDIGe_2_. Thermal ellipsoids are depicted at the 50 % probability level, and hydrogen atoms are omitted for clarity. Selected bond lengths [Å], bond angles [°] and dihedral angles [°] with estimated standard deviations: Ge(1)−C(4) 1.971(4); Ge(1)−C(8) 1.973(4); Ge(1)−C(26) 1.956(4); Ge(1)−C(28) 1.948(5); C8 Ge(1) 1.973(4); C(1)−O(1) 1.224(5); C(12)−O(2) 1.218(5); C(1)−N(1) 1.394(6); C(12)−N(1) 1.402(5); C(13)−N(1) 1.508(5); C(4)−Ge(1)−C(8) 89.74(16); C(26)−Ge(1)−C(4) 109.87(17); C(26)Ge(1)−C(8) 114.86(17); C(28)−Ge(1)−C(4) 112.79(19); C(28)−Ge(1)−C(8) 115.85(19); C(28)−Ge(1)−C(26) 111.8(2); O(1)−C(1)−N(1) 120.9(4); C(1)−N(1)−C(12) 125.2(3); C(1)−N(1)−C(13) 118.0(3); C(12)−N(1)−C(13) 116.8(4); C(3)−C(4)−Ge(1) 135.9(3); C(9)−C(8)−Ge(1) 135.5(3); Ge(1)−C(8)−C(9)−C(10) 179.9(3).

The molecule PDICl_4_ was synthesized according to procedures published by Holman et al.^[23]^ Following procedures similar to those performed by Ma et al.,^[24]^ the target compounds, silyl (PDISi, PDISi_2_) and germyl (PDIGe, PDIGe_2_) substituted perylene diimides, were formed in a palladium catalyzed cross‐coupling reaction with PDICl_4_ and either H_2_SiEt_2_ or H_2_GeEt_2_ (Figure 1). At the beginning of our investigations, we screened various palladium derivatives and various ligands. The best yields were obtained by the system Pd(OAc)_2_ and P(*t*Bu)_3_. In all cases we used an excess of an auxiliary base (DIPEA=EtN(*i*Pr)_2_). After the reaction the mono‐ and disubstituted PDIs were separated via column chromatography. For PDISi, PDISi_2_, PDIGe and PDIGe_2_ analytical data are consistent with the proposed structures (Figures S3–S12 in the Supporting Information). For further proof, MALDI‐TOF MS measurements were carried out (Figures S14–S17). For PDIGe_2_, single crystals suitable for X‐ray structure analysis could be grown in chloroform at −30 °C. The crystal structure thereof is depicted in Figure 2. PDIGe_2_ crystallized in the monoclinic space group *P*2(1)*c* with a unit cell containing four molecules. The packing in the single crystal (Figure S18) shows the presence of two orientations in the PDIGe_2_ crystal and stacking distances of 12.172 Å and 23.356 Å, measured between adjacent molecules along the edges of the unit cell.

However, the X‐ray analysis shows that the π‐conjugated perylene skeleton of PDIGe_2_ is planar, indicating that the substitution on the bay position did not alter the planarity of the perylene core. The bond lengths of germanium‐carbon (Ge−C) bonds (1.971(4), 1.973(4), 1.956(4) Å) match the literature known distances (1.98±0.03 Å).^[25]^ All other bond lengths (C−O 1.218(5)–1.218(5) Å; C−N 1.394(6)–1.508(5) Å) are similar to other literature known PDIs (C−O 1.214 Å;^[26]^ C−N 1.392(6)–1.457(6) Å).^[27]^ In addition, the values of the dihedral angles were in agreement with the values obtained by DFT calculations.

### DFT calculations

DFT calculations of all four acceptor molecules were performed at the B3LYP/6‐31 d,p level of theory with an empirical dispersion correction using the Gaussian 16 program package.^[28]^ The molecules were geometrically optimized under vacuum conditions to assess whether their planarity was distorted when introducing silicon and germanium to their structure. The results showed that one or two additional Si atoms have no impact on their planarity. PDISi and PDISi_2_ maintained a dihedral angle (derived from Si/Ge and the adjacent carbon atoms) of approximately 179.7° and 179.6°, respectively. The larger atom size of Ge changed the angles more, having 179.0° in PDIGe and 178.0° in PDIGe_2_. This shows that the planarity of the molecule is not significantly affected, even if large atoms are introduced in the bay region. These results were also confirmed by the crystal structure of PDIGe_2_, which shows no distortion of the planarity by Ge atoms (with dihedral angles of 179.2° and 179.9°; Figure 2).

**Figure 3:**
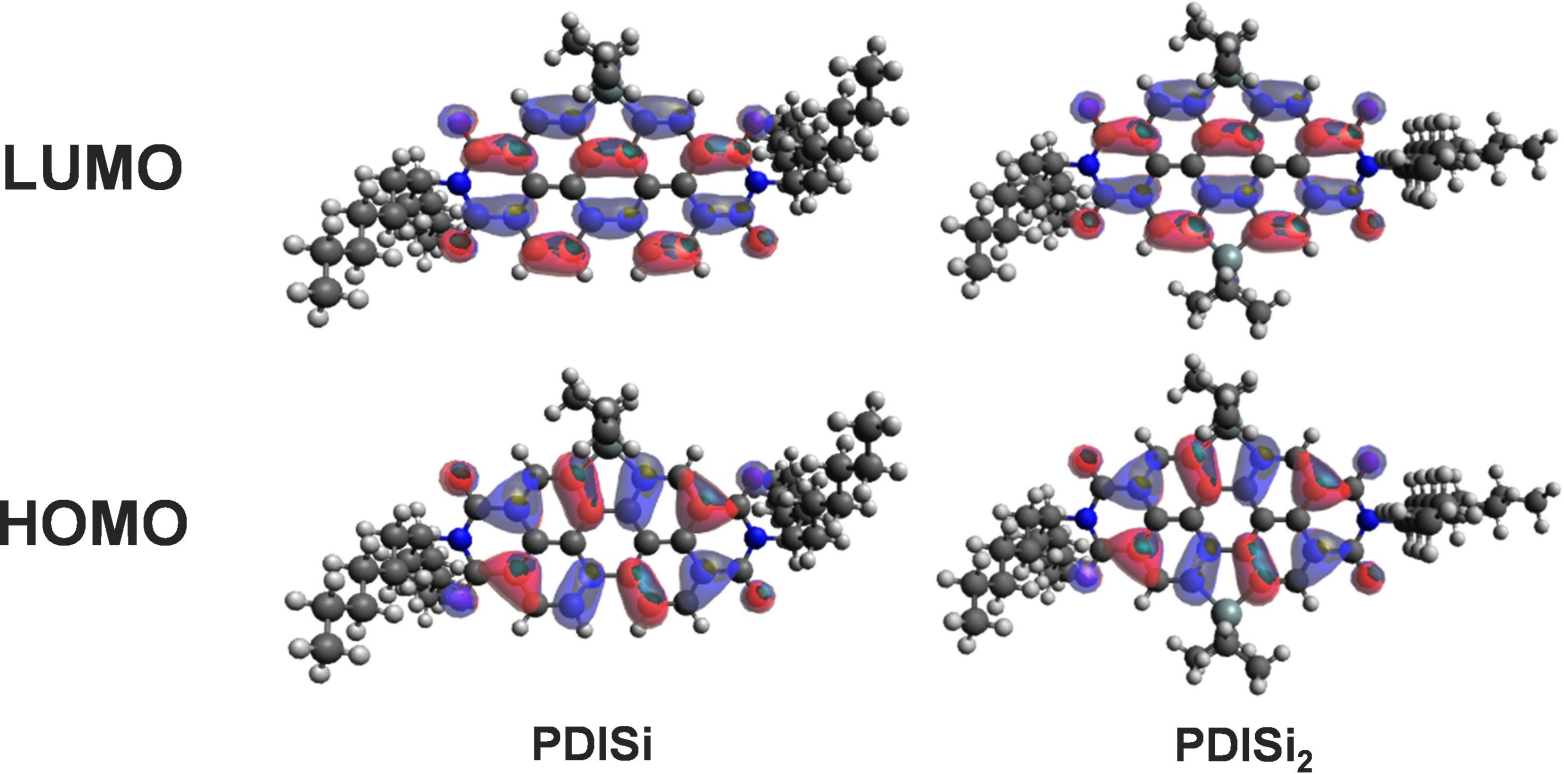
Calculated electron density distribution of HOMOs and LUMOs of PDISi and PDISi_2_.

Furthermore, the HOMO/LUMO energy levels were calculated to be −6.10/−3.66, −6.00/−3.58, −6.09/−3.65 and −5.99/−3.57 for PDISi, PDISi_2_, PDIGe and PDIGe_2_, respectively (Figure S23). All molecules have low lying energy levels, slightly higher for the disubstituted than for the monosubstituted molecules. These results corroborate the findings of Ma et al.,^[24]^ who introduced silyl PDIs with varying side chains on the imide position. In addition, they show no distribution of the HOMO energy levels over the sp^3^‐hybridized semimetal and only small contributions of the Si−C bond to the LUMO, as found in our silyl and germyl acceptors (Figures 3 and S24). In contrast, in S and Se bay‐annulated PDIs, HOMOs and LUMOs are distributed over the heteroatoms due to the involvement of the chalcogen lone pairs.^[29]^

### Optical and electrochemical properties

**Figure 4:**
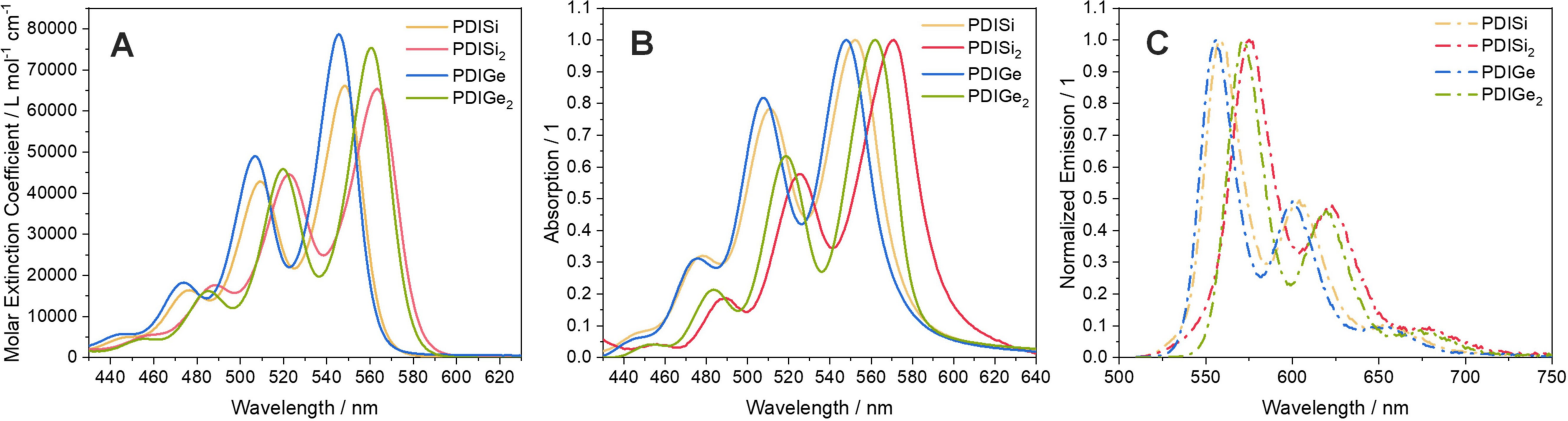
A) Absorption spectra of all acceptors in CHCl_3_ solution. B) Absorption spectra of all acceptors in thin films coated on glass substrates. C) Fluorescence spectra of all acceptors in CHCl_3_ solution.

The absorption spectra of all compounds in chloroform solution are shown in Figure 4A. All spectra show a broad absorption over 400–600 nm with the typical absorption pattern for PDIs.^[30]^ The absorption maxima of PDISi and PDIGe as well as PDISi_2_ and PDIGe_2_ are almost at identical wavelengths. However, compared to the unmodified PDI molecule with an absorption maximum at 526 nm (Figure S21), the absorption maxima of our acceptors are red‐shifted (Table 1), most likely due to the contribution of the Si−C/Ge−C bond in the excited state, as shown by DFT computations. Therefore, the red‐shift is enhanced upon the addition of a second silicon or germanium atom. The molar extinction is over 60 000 L mol^‐1^ cm^‐1^ for all acceptors and even higher (>70 000 L mol^−1^ cm^−1^) for PDIGe and PDIGe_2_.

**Table 1:** Optical and electrical properties of compounds PDISi, PDISi_2_, PDIGe and PDIGe_2_.

	Abs. max.^[a]^ [nm]	*ϵ* [L mol^‐1^ cm^−1^]	Em. max. [nm]	QY_rel_ [%]	Lifetimes [ns]	Abs. max.^[b]^ [nm]	*E*_g_^opt^ [eV]	HOMO^[c]^ [eV]	LUMO^[c]^ [eV]	HOMO^[d]^ [eV]	LUMO^[d]^ [eV]
PDISi	548	6.3×10^5^	558	≥99	4.69	552	2.15	−6.73	−4.58	−6.10	−3.66
PDISi_2_	563	6.7×10^5^	575	93	5.74	571	2.08	−6.55	−4.47	−6.00	−3.58
PDIGe	546	7.9×10^5^	556	≥99	4.51	548	2.17	−6.47	−4.30	−6.09	−3.65
PDIGe_2_	561	7.4×10^5^	571	96	5.46	562	2.13	−6.52	−4.39	−5.99	−3.57

[a] In chloroform solution. [b] In thin films. [c] HOMO energy levels derived from CV measurements as described in the supporting information; LUMO levels calculated from the HOMO and the optical band gap. [d] HOMO and LUMO energy levels derived from DFT computations.

In thin films, the absorption of these compounds is further red‐shifted, more so for the silyl than for the germyl acceptors (Figure 4B). The optical band gap was obtained from the absorption onset and is highest in PDIGe (2.17 eV), followed by PDISi (2.15 eV) and PDIGe_2_ (2.13 eV) and hence the lowest in PDISi_2_ (2.08 eV).

Fluorescence properties were also determined in chloroform solution, including the relative quantum yield and fluorescence lifetimes. The fluorescence bands are depicted in Figure 4C and the corresponding values are listed in Table 1. All compounds displayed the same spectral pattern and a Stokes shift of approximately 10 nm. The monosubstituted compounds had the highest relative quantum yield, approaching 100 %, followed by PDIGe_2_ with 96 % and PDISi_2_ with 93 %. The fluorescence lifetimes of the asymmetric compounds are >4.5 ns and even longer for the symmetric ones with ~5.5 ns. The longer lifetimes of the excited state should be beneficial for the charge separation at the donor–acceptor interface in solar cell devices.^[31]^

**Figure 5:**
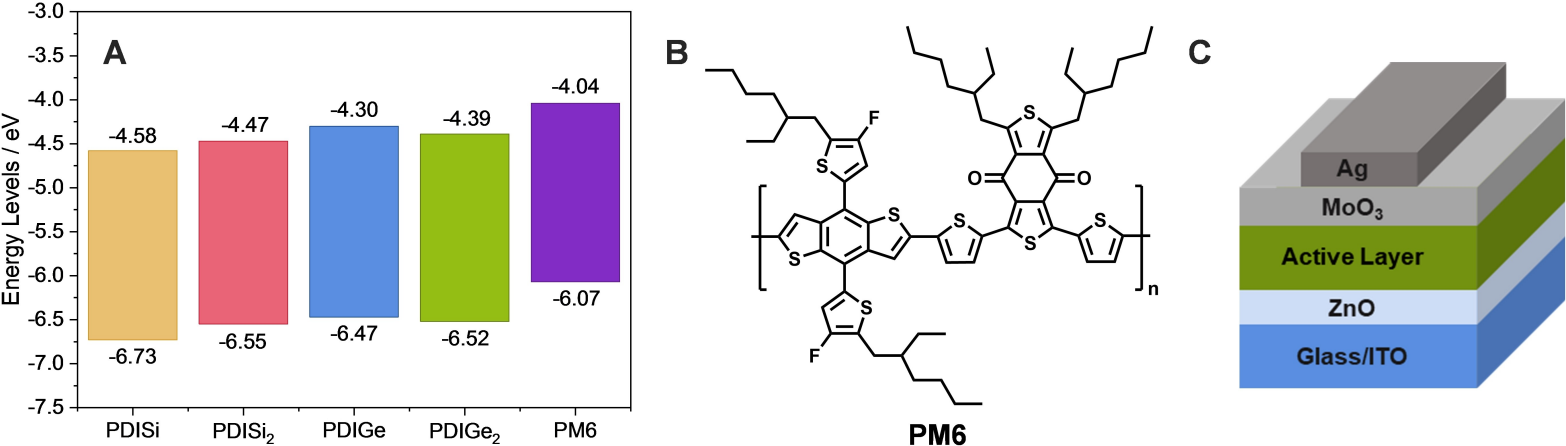
A) HOMO and LUMO energy levels of the investigated absorber materials. B) Structure of the polymeric donor PM6. C) Device structure of the solar cells.

The electrochemical properties were assessed by cyclic voltammetry (Figure S25). Thin films of the compounds were drop casted on a Pt disk electrode from chloroform solutions. From the oxidation onset, the HOMO energy level was calculated (see the Supporting Information, Equation (1)). The LUMO energy levels were derived from the HOMO and the optical band gap of the respective materials. The silyl compounds show slightly lower HOMO energy levels than the germyl‐based compounds (Table 1). The values derived from the DFT computations are slightly upshifted when compared with those derived from CV measurements, as expected because the theoretical values were calculated under vacuum conditions and the experimental data include other phenomena such as redox kinetics and solvent effects. Figure 5A shows a graphical representation of the experimental energy levels of all acceptors and the donor molecule PM6. The energy levels of the synthesized acceptors match well with the donor, indicating the potential for a high *V*_OC_ in OPVs.

### Organic solar cells

**Figure 6:**
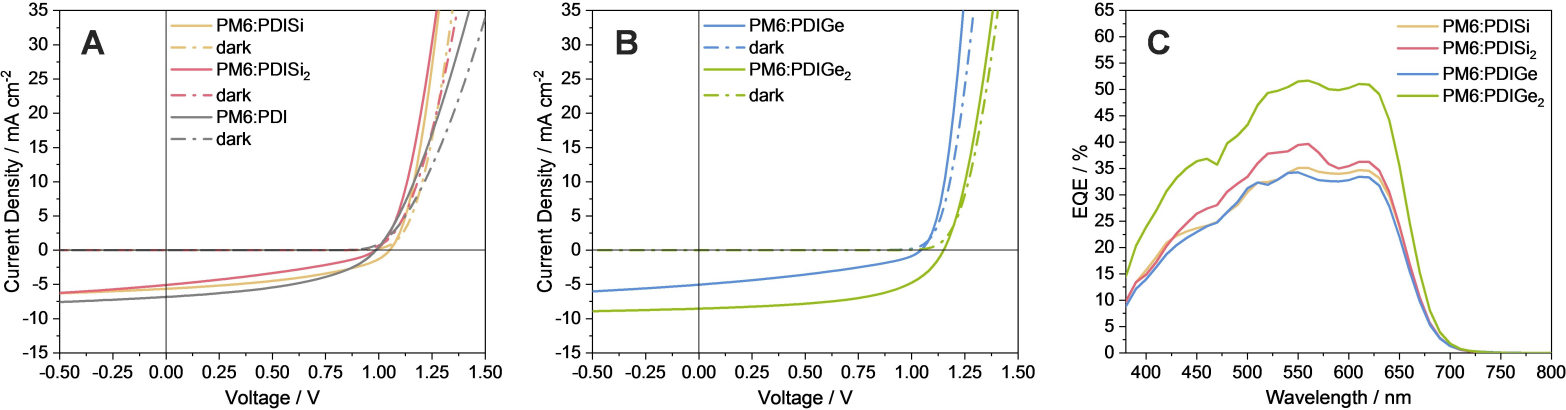
JV characteristics of solar cells containing A) PM6 : PDI, PM6 : PDISi and PM6 : PDISi_2_ and B) PM6 : PDIGe and PM6 : PDIGe_2_ absorber layers. C) External quantum efficiency (EQE) spectra of solar cells based on the PDI acceptors combined with PM6.

To evaluate their photovoltaic properties, the non‐fullerene acceptors were applied in combination with the conjugated polymer PM6 (depicted in Figure 5) in bulk heterojunction organic solar cells. The solar cells were prepared using an inverted device structure with the layer sequence ITO/ZnO/active layer/MoO_3_/Ag (Figure 5C). Based on our experience from a previous study^[32]^ and on several optimization steps, solar cells prepared with a D : A weight ratio of 1 : 1 (total concentration: 20 mg mL^‐1^), an active layer with a thickness of 60–80 nm and annealing at 140 °C led to the highest PCEs. The JV curves, EQE spectra and characteristic data of the solar cells are presented in Figure 6 and Table 2, respectively.

**Table 2:** Characteristic parameters of solar cells with the silicon‐ and germanium‐based PDI‐derivatives as non‐fullerene acceptors in combination with the donor polymer PM6; all solar cells were annealed at 140 °C and have an active layer thickness of 60 nm; mean values and standard deviations are calculated from ten solar cells (best values in brackets).

Active layer	*V*_OC_ [V]	I_SC_ [mA cm^−2^]	FF [%]	PCE [%]	*α*/n	Electron mobility [cm^2^ V^−1^ s^−1^]	Hole mobility [cm^2^ V^−1^ s^−1^]
PM6 : PDI	0.99±0.01 (0.99)	6.67±0.28 (6.81)	44.2±0.53 (45.0)	2.88±0.14 (2.99)	–	–	–
PM6 : PDISi	1.04±0.01 (1.06)	5.31±0.20 (5.63)	42.6±0.79 (43.2)	2.33±0.09 (2.55)	0.98/1.16	1.08×10^−6^±2.90×10^−7^	2.53×10^−4^±4.36×10^−5^
PM6 : PDISi_2_	0.98±0.01 (0.97)	4.77±0.20 (5.07)	35.3±0.36 (35.3)	1.63±0.06 (1.73)	0.92/1.16	1.51×10^−7^±2.02×10^−8^	3.30×10^−4^±2.80×10^−5^
PM6 : PDIGe	1.04±0.01 (1.03)	4.67±0.22 (5.02)	36.2±0.54 (37.0)	1.74±0.11 (1.92)	0.96/1.23	6.28×10^−7^±9.27×10^−8^	2.94×10^−4^±2.41×10^−5^
PM6 : PDIGe_2_	1.14±0.01 (1.16)	8.04±0.35 (8.53)	54.6±1.51 (55.1)	4.97±0.24 (5.38)	1.00/1.12	1.54×10^−5^±1.33×10^−6^	1.59×10^−4^±8.82×10^−6^

The PM6 : PDIGe_2_ blend showed the best photovoltaic performance, with a maximum efficiency of 5.38 %, thus ranking among the top‐10 best monomeric PDI molecules by now.^[14]^ This value is also significantly higher than the PCE of devices containing the same PDI without semimetals, where we reached values of up to 3 % (Table 2 and Figure 6). The high *V*_OC_ of 1.16 V and FF of 55.1 % of PM6 : PDIGe_2_‐based solar cells account for their good overall performance. The other three acceptors yielded a *V*_OC_ of approximately 1 V, and for PDISi_2_ and PDIGe, much lower fill factors and *J*_SC_ values are derived. The PM6 : PDISi blend reached approximately half of the efficiency of PM6 : PDIGe_2_, with a slightly higher *J*_SC_ and a higher FF than PDISi_2_ and PDIGe‐based solar cells. In turn, PDISi_2_ and PDIGe‐based solar cells showed PCE values of only 1.6 and 1.7 %, respectively, which was unexpected, as the asymmetric molecules have similar optical properties, as have the symmetric ones. In addition, we also prepared solar cells with PBDB‐T instead of PM6 as donor, finding similar results (Figure S27, Table S2). The PBDB‐T : PDIGe_2_ blend showed the highest PCE of up to 4.95 %, followed by the PBDB‐T : PDISi blend (2.53 %) and the other two absorber layer blends, with PCEs lower than 2 % similar to their analogs with PM6 as donor.

By performing light intensity dependent measurements, we gained insights into recombination processes in the devices. The *α* value (power law dependence of *J*_SC_ vs. *I* ^*α*^, *I*: light intensity) describes the tendency for trap‐assisted recombination under short circuit conditions in the absorber layer (for *α*<1) and almost no recombination at short circuit conditions when *α* is approaching 1. Here, the PM6 : PDIGe_2_ blend had an *α* value of 1, which decreased to 0.92 in the following order: PM6 : PDISi, PM6 : PDIGe, PM6 : PDISi_2_, in line with the efficiencies of the devices (Table 2). In addition, we determined the ideality factor. The ideality factor describes whether mono‐ or bimolecular recombination prevail in the cell (*n* values near 1 indicate bimolecular recombination; and values close to 2 indicate monomolecular or trap assisted recombination). For PM6 : PDIGe_2_ based solar cells, an ideality factor of 1.12 is found which increased when using PDISi and PDISi_2_ (1.16 each) and PDIGe (1.23) as acceptors, thus showing a slight tendency towards the prevalence of trap‐assisted recombination.

The EQE spectra (Figure 6C) reveal an onset slightly above 700 nm, which matches well with the absorption onset of PM6. The local maxima at around 640 nm stem from the PM6 contribution. The maxima visible between 480 and 570 nm are shifting and match with the positions of the maxima in the absorption spectra of the respective PDI‐based acceptor (Figure 4C) overlapping with PM6. In accordance with the JV characterizations, the symmetric acceptor PDIGe_2_ yields the solar cells with the highest EQE values, followed by PDISi_2_, PDISi and PDIGe. Moreover, the J_SC_s extracted from the EQE spectra (PM6 : PDISi: 5.0 mA cm^‐2^, PM6 : PDISi_2_: 5.5 mA cm^‐2^, PM6 : PDIGe: 4.9 mA cm^‐2^ and PM6 : PDIGe_2_: 7.5 mA cm^‐2^) correlate well with the *J*_SC_ values derived from the JV curves.

Moreover, the electron and hole mobilities in the absorber layer were determined by the space charge limited current (SCLC) method using hole‐ and electron‐only devices. The device architectures, the recorded data as well as the data analysis are shown in Figure S28, and the obtained mobilities are listed in Table 2. Despite the similar hole mobilities in all four absorber layers, the electron mobilities considerably deviated, and the electron mobility was more than 10 times higher in the PM6 : PDIGe_2_ blend than in the PM6 : PDISi blend, which had the second highest PCE. The PM6 : PDIGe and PM6 : PDISi_2_ samples had an even lower electron mobility, which may explain the low efficiencies of these materials in solar cells and their low *α* values. Imbalanced charge carrier mobilities were also expected to lead to a more pronounced buildup of space charge in solar cells, lowering the fill factors. Consequently, the PM6 : PDIGe_2_ blend with the highest electron mobility has the highest fill factors and *α* values closest to 1.

### GIWAXS characterizations

**Figure 7:**
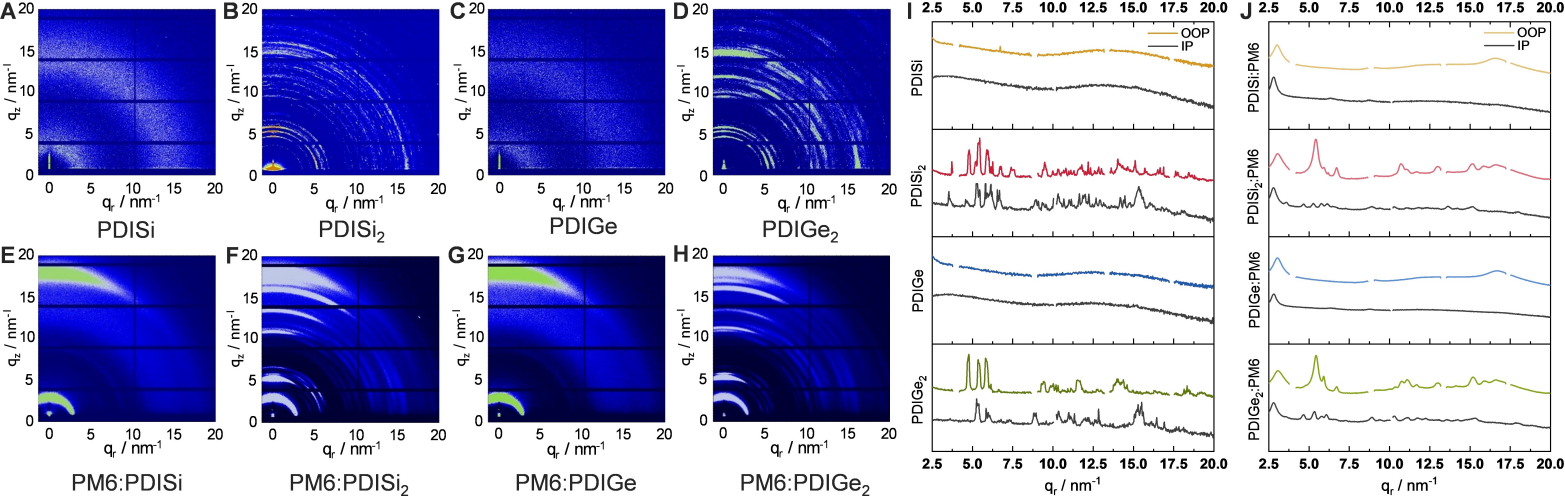
2D GIWAXS images of A) PDISi, B) PDISi_2_, C) PDIGe, D) PDIGe_2_, E) PM6 : PDISi, F) PM6 : PDISi_2_, G) PM6 : PDIGe, H) PM6 : PDIGe_2_ and the corresponding line cuts in plane (gray) and out of plane (colored) of I) the acceptors and J) the active layer blends.

To learn about the crystallinity and orientation of the acceptor materials and their blends with PM6 and PBDB‐T, we conducted 2D GIWAXS measurements. The acceptors and donor : acceptor blends were drop casted from chlorobenzene solutions on silicon substrates and were subsequently annealed at 140 °C for 10 min. When comparing mono‐ with disubstituted perylenes, the disubstituted perylenes reveal a distinct crystallinity, albeit without a preferred orientation with respect to the substrate in the thin films, as shown in the GIWAXS images (Figure 7A–D). The PDISi_2_ and PDIGe_2_ films show a significant peak at 14.2 nm^−1^ and a smaller one at 15.1 nm^−1^ revealing π‐π stacking distances of 0.44 and 0.42 nm. Moreover, the distinct peaks at lower q‐values indicate a highly ordered stacking with prominent distances of 1.31, 1.16 and 1.08 nm. No significant differences in the stacking distances of the silicon and germanium‐based compounds are observed. In the packing of the single crystal of PDIGe_2_, the same stacking distance of around 1.2 nm is found, but the π‐π stacking distance of 0.42 nm is not observed. This is most likely due to a faster crystallization of the molecules in thin films leading to a slightly changed molecular packing and the additional annealing, which enhances the overall crystallinity. Moreover, PDIGe_2_ reveals a mixed face‐on and edge‐on orientation with respect to the substrate in the pristine thin film (Figure 7D), while in the blend with PM6 (Figure 7H), no features indicating an edge‐on orientation are observed in the GIWAXS data. The GIWAXS images of the PDISi and PDIGe samples display only vague semi‐circle‐like features around 3–3.5 nm^−1^ and 12.5–17 nm^‐1^, corresponding to lamellar stacking distances of 2.1–1.8 nm and π‐π stacking distances of 0.37–0.5 nm, respectively. These stacking distances are similar to those recorded in our previous analysis of other perylene acceptors.^[32]^

Combined with the donor polymer PM6, the acceptors show a more preferred face‐on orientation in the active layer, which is advantageous for the vertical charge transport in the solar cell. The pronounced features in the PM6 : PDISi and PM6 : PDIGe blends (at 3.05 and 16.6 nm^−1^ corresponding to a *d*‐spacing of 2.06 and 0.38 nm, out of plane) stem from the polymer phase, whereas the blurrier section below 15.5 nm^−1^ can be ascribed to the acceptors, as they lie in the same range as the outer semi‐circle present in the pristine acceptor films (Figure 7a,c). For lamellar stacking, only the feature stemming from PM6 is recognized.

In the PM6 : PDISi_2_ and PM6 : PDIGe_2_ blend films, distinct diffraction peaks are observed in addition to the ones stemming from the polymer. Interestingly, only one intense lamellar stacking peak at 5.40 nm^‐1^ (real space distance: 1.16 nm) is observed in the blends containing the disubstituted PDI‐derivatives, which indicates a reduced 2D order in the acceptor phase in the PM6 : PDISi_2_ and PM6 : PDIGe_2_ blends. The π‐π stacking distance of PDISi_2_ and PDIGe_2_ in the blends (~0.41 nm) is slightly narrower than that of the pristine films.

As in the characterization of the photovoltaic properties, we performed a GIWAXS analysis of both PBDB‐T‐ and PM6‐acceptor blends, finding similar results, with only small differences in the orientation of PBDB‐T : PDISi_2_ and PBDB‐T : PDIGe_2_ films (Figure S19).

### Microscopy

To further analyze the absorber layers, we acquired bright field optical microscopy images (see Figure S20) which revealed a homogeneous coating in all four absorber layers. Dark‐field images also support the GIWAXS results and indicate that the crystallinity of the PM6 : PDISi_2_ and PM6 : PDIGe_2_ thin films is significantly higher than that of their monosubstituted analogs.

**Figure 8:**
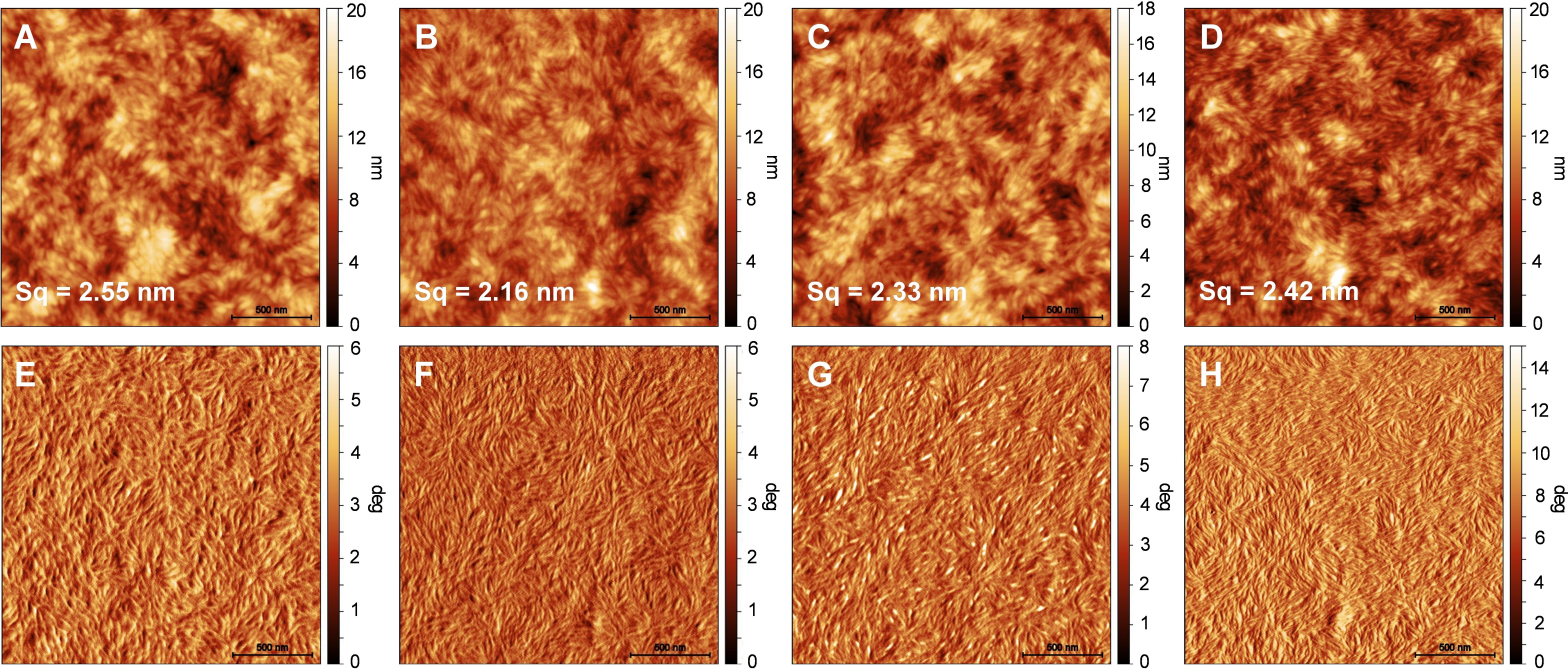
AFM height and phase images (size: 2 μm×2 μm) of the active layer of PM6:acceptor‐based solar cell devices. A), E) PM6 : PDISi, B), F) PM6 : PDISi_2_, C), G) PM6 : PDIGe, D), H) PM6 : PDIGe_2_. The phase angle ranges between 0–6° or 0–8° in E–G and between 0–15° in H (PM6 : PDIGe_2_).

Moreover, topography and phase AFM images were measured to investigate the surface morphology and phase separation of the bulk heterojunction films. The images in Figure 8 reveal elongated structures in the topography as well as the phase images. The fibrillar structures likely correspond to the PDI acceptor phase and they are slightly thinner in the disubstituted than in the monosubstituted PDI derivatives. The PM6 : PDIGe_2_ blend, which has the highest crystallinity based on the GIWAXS measurements, and the highest photovoltaic performance, forms the finest donor–acceptor phase separation within this set of compounds. Moreover, the higher phase contrast (the phase angle range is 0–15° in Figure 8H compared to 0–6° or 0–8° for the other blends) indicates that the phase separation is significantly more defined in the PM6 : PDIGe_2_ blend, which could be an indication for higher phase purities.

### Transient absorption spectroscopy

**Figure 9:**
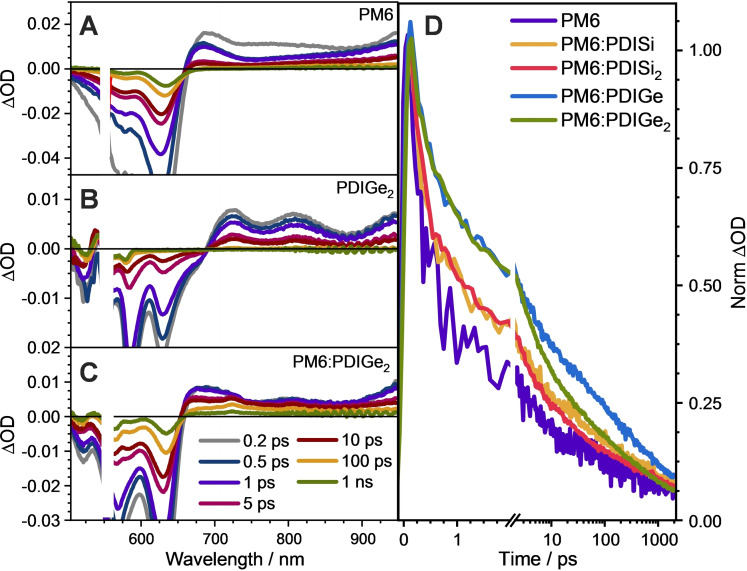
Difference absorption spectra of pristine A) PM6 and B) PDIGe_2_ as well as C) PM6 : PDIGe_2_ blend films at several delay times. D) GSB recovery of pristine PM6 and all the studied blends probed at 630 nm (*λ*_ex_=550 nm, *E*~80 nJ). The results for the pristine and blend films of the other PDI derivatives can be found in the Supporting Information.

To gain further insight into the performance of these PDI derivatives as non‐fullerene acceptors, fs‐TAS was performed on pristine and blend films with PM6 (Figures 9 and S29). Difference absorption spectra recorded at several delay times for PM6 (Figure 9A) show a positive excited absorbance (ESA) with a band at around 700 nm, which extends to the infrared region. The negative region between approx. 500 and 650 nm matches the ground state absorbance and was therefore assigned to the ground state bleach absorption (GSB). The PM6 photoexcited states spectrum and lifetimes are in line with previous reports on PM6 localized excitons and intra‐moiety excited states.[^33^, ^34^] The pristine PDI derivatives (Figure 9B and S29A–D) show a single positive ESA feature with a multiband shape. In addition to their GSB features, the pristine PDI derivatives also show large negative bands associated with the stimulated emission (SE) at 606, 624, 600 and 627 nm for PDISi, PDISi_2_, PDIGe and PDIGe_2_, respectively.

The TA spectra of the blend films (Figure 9C and S29E–H), however, reveal that the 700 nm band associated with PM6 excitons becomes featureless and broader at longer lifetimes. This feature is associated with the charge‐separated states of PM6 and the PDI derivatives.[^34^, ^35^] In addition, these blends also show the GSB corresponding to PM6 (with a negative band at 630 nm) and the PDI derivatives (with bands matching the absorbance of the different PDIs). The negative band at 630 nm cannot be associated with the SE of the PDIs as the position of this band is at the same wavelength irrespective of the acceptor used (Figure 4). Moreover, the PDI emission should be largely quenched when blended with PM6. However, minor contributions of the SE of the PDIs cannot be fully excluded. Still, the band at 630 nm is a good candidate to follow the kinetics of PM6 related species due to the strong GSB absorption of PM6 and to avoid species overlapped with ESA in the positive region of the TA spectra.

The normalized PM6 bleach kinetics, probed at 630 nm, are shown in Figure 9D. As seen in the pristine sample, the PM6 ground state recovers with a multiexponential decay with a fast first component of around 0.3 ps. This indicates that a large portion of PM6 excitons quickly decay to the ground state in the first picosecond and this is partly mitigated when the material is blended with an acceptor as the exciton is transformed into longer lived species. The first fast phase of GSB is prolonged in the blend samples compared to the neat PM6 film, but the excited state lifetime extension does not reach the values of other materials reported in the literature.[^34^, ^36^] Accordingly, the injection from the PM6 donor to the PDI acceptor is not as efficient as in other acceptors, thus explaining the moderate *J*_SC_ values of the devices. Moreover, the injection efficiency is not directly associated with the differences of the LUMO level in the materials (Figure 5). These materials follow a different trend primarily determined by the heteroatom, where the injection to the Ge derivatives seems to be more efficient than to the Si derivatives.

At longer timescales (>10 ps), where the charges within the PM6 phase are expected to geminately recombine, the trend does change: PDIGe_2_ shows the fastest decay to ground‐state. These faster kinetics are in agreement with the higher crystallinity and hole mobility of PDIGe_2_ blends, as discussed above. Despite, faster kinetics could be generally associated with a decrease in device performance, large mobilities nevertheless can enable charge extraction before recombination, increasing the overall efficiency.^[37]^ Thus, the increased crystallinity should be the reason for the increased FF of the most efficient PM6 : PDIGe_2_ device.

## Conclusions

Semimetal‐containing asymmetric and symmetric non‐fullerene acceptors based on a monomeric PDI unit can be prepared in an efficient three‐step synthesis. These acceptors contain one or two silicon or germanium atoms introduced in the bay position of the PDIs. All four materials show a red‐shift in absorption compared to an unmodified PDI, with a larger shift for the disubstituted compounds. While the four molecules differ only minimally in their optical properties, they differ significantly in their performance as non‐fullerene acceptors in inverted solar cells. The best performing perylene is, by far, PDIGe_2_ combined with the polymer PM6, reaching a PCE of over 5 %, primarily thanks to the relatively high electron mobility in the absorber layers and high crystallinity of PDIGe_2_. In addition, fs‐TAS analyses show that injection from the donor PM6 is more efficient for the Ge‐containing acceptors, and the higher crystallinity in the PM6 : PDIGe_2_ blend is beneficial for charge extraction, thus increasing the overall device performance. The PCE values of the PM6 : PDIGe_2_ blend are among the highest recorded with monomeric perylene acceptors and show that a reasonable efficiency can be achieved with simple non‐fullerene acceptors. Overall, this study demonstrates that introducing semimetals such as Si and Ge to perylene diimides can be beneficial for increasing the performance of perylene acceptors. This strategy can be extended to a variety of perylene‐based non‐fullerene acceptor designs, which might lead to high‐performing, simple and inexpensive acceptors for organic photovoltaics.

## Experimental Section

### Synthesis

*5,6,12,13‐Di(diethylsilyl)‐2,9‐di(tridecan‐7‐yl)anthra[2,1,9‐def* : *6,5,10‐d′e′f′]diisoquinoline‐1,3,8,10(2*H*,9*H*)‐tetraone (PDISi) and 5,6,–diethylsilyl‐2,9‐di(tridecan‐7‐yl)anthra[2,1,9‐def* : *6,5,10‐d′e′f′]diisoquinoline‐1,3,8,10(2H,9H)‐tetraone (PDISi_2_)*: 1.02 mL Et_2_SiH_2_ (7.84 mmol), an excess of EtN(*i*Pr)_2_ and a catalytic amount (one spatula tip) of Pd(OAc)_2_ and P(*t*Bu)_3_ were added to a solution of 1.00 g of PDICl_4_ (1.12 mmol) in 10.0 mL toluene and stirred for 48 h at 100 °C. The reaction mixture was allowed to cool down to room temperature and the solvent was removed in vacuum. The dark purple residue was purified by column chromatography with a gradient elution of *n*‐heptane and dichloromethane to obtain 0.317 g (33.7 %) of PDISi (dark red solid) and 0.260 g (25.1 %) of PDISi_2_ (dark pink solid).

PDISi: ^1^H NMR (200 MHz, CD_2_Cl_2_): *δ*=8.84 (s, 2H, aryl *H*), 8.57 (s, 4H, aryl *H*), 5.27–5.11 (sept, 2H, C*H*), 2.43–2.10 (m, 4H, C*H*_2_), 1.95–1.72 (m, 4H, C*H*_2_), 1.35‐1.20 (m, 36H, C*H*_2_), 1.09 (s, 3H, C*H*_3_), 1.06 (s, 3H, C*H*_3_), 0.87–0.79 ppm (m, 12H, C*H*_3_). ^13^C NMR (76 MHz, CDCl_3_): *δ*=165.61, 164.73, 164.57, 163.66 (*C*O), 146.91, 136.95, 135.09, 132.55, 130.32, 124.70, 123.18 (aryl *C*), 54.77 (*C*H), 32.56, 31.87, 29.35, 27.04, 22.69, 14.13 (*C*H_2_), 7.80, 2.82 ppm (*C*H_3_). ^29^Si NMR (40 MHz, CDCl_3_): *δ*=12.43 ppm (s, ‐*Si*Et_2_). UV/Vis (CHCl_3_): *λ*_max_ (*ϵ*)=548 (63 048 mol^−1^ dm^3^ cm^−1^); fluorescence (CHCl_3_): *λ*_ex_=495 nm; *λ*_em_=558 nm; HRMS (MALDI‐TOF): *m/z* calcd for C_54_H_70_N_2_O_4_Si: 838.5105 [*M*]; found: 838.5114; FTIR (cm^−1^): *ṽ*=2955, 2925 and 2856 (aliphatic C−H), 1693 and 1656 (C=O), 1588 (C=C), 1457 and 1400 (aliphatic C−H), 1295 (C−N).

PDISi_2_: ^1^H NMR (200 MHz, CD_2_Cl_2_): *δ*=8.86–8.70 (d, 4H, aryl *H*), 5.24–5.16 (sept, 2H, C*H*), 2.30–2.17 (m, 4H, C*H*_2_), 1.88–1.75 (m, 4H, C*H*_2_), 1.35–1.16 (m, 40H, C*H*_2_), 1.10 (s, 3H, C*H*_3_), 1.07 (s, 3H, C*H*_3_), 0.98 (s, 3H, C*H*_3_), 0.95 (s, 3H, C*H*_3_), 0.86–0.78 ppm (t, 12H, C*H*_3_). ^13^C NMR (76 MHz, CDCl_3_): *δ*=165.79, 164.73 (*C*O), 147.38, 137.02, 136.46, 130.60, 121.97 (aryl *C*), 54.82 (*C*H), 32.72, 31.93, 29.43, 27.12, 22.75, 14.18 (*C*H_2_), 7.89, 3.19 ppm (*C*H_3_). ^29^Si NMR (40 MHz, CDCl_3_); *δ*=15.98 (s, −*Si*Et_2_) ppm. UV/Vis (CHCl_3_): *λ*_max_ (*ϵ*)=563 (66 898 mol^−1^ dm^3^ cm^−1^); fluorescence (CHCl_3_): *λ*_ex_=495 nm; *λ*_em_=575 nm; HRMS (MALDI‐TOF): *m/z* calcd for C_58_H_78_N_2_O_4_Si_2_: 922.5500 [*M*]; found: 922.5505; FTIR (cm^−1^): *ṽ*=2956, 2925, 2873 and 2856 (aliphatic C−H), 1688 and 1654 (C=O), 1590 (C=C), 1457 and 1418 (aliphatic C−H), 1278 (C−N).

*5,6,12,13‐Di(diethylgermyl)‐2,9‐di(tridecan‐7‐yl)anthra[2,1,9‐def:6,5,10‐d′e′f′]diisoquinoline‐1,3,8,10(2*H*,9*H*)‐tetraone (PDIGe) and 5,6,–diethylgermyl‐2,9‐di(tridecan‐7‐yl)anthra[2,1,9‐def:6,5,10‐d′e′f′]diisoquinoline‐1,3,8,10(2H,9H)‐tetraone (PDIGe_2_)*: 1.53 mL Et_2_GeH_2_ (7.84 mmol), an excess of EtN(iPr)_2_ and a catalytic amount (one spatula tip) of Pd(OAc)_2_ and P(*t*Bu)_3_ were added to a solution of 1.00 g of PDICl_4_ (7.84 mmol) in 10.0 mL toluene and stirred for 48 h at 100 °C. The reaction mixture was allowed to cool down to room temperature and the solvent was removed in vacuum. The dark purple residue was purified by column chromatography with a gradient elution of *n*‐heptane and dichloromethane to obtain 0.321 g (32.4 %) of PDIGe (red solid) and 0.290 g (25.5 %) of PDIGe_2_ (dark pink solid).

PDIGe: ^1^H NMR (300 MHz, CDCl_3_): *δ*=8.90–8.80 (d, 2H, aryl *H*), 8.70–8.55 (m, 4H, aryl *H*), 5.26–5.17 (m, 2H, C*H*), 2.30–2.19 (m, 4H, C*H*_2_), 1.92–1.79 (m, 4H, C*H*_2_), 1.51–1.42 (m, 4H, C*H*_2_), 1.30‐1.18 (m, 32H, C*H*_2_), 1.17–1.13 (d, 6H, C*H*_3_), 0.84–0.80 ppm (t, C*H*_3_). ^13^C NMR (100.6 MHz, CDCl_3_): *δ*=165.77, 164.92, 164.76, 163.86 (CO), 145.79, 140.41, 136.06, 135.09, 132.25, 131.48, 130.03, 125.19, 123.07 (aryl *C*), 54.87 (*C*H), 32.64, 31.92, 29.41, 27.11, 22.75, 14.19 (*C*H_2_), 9.62, 6.25 ppm (*C*H_3_). UV/Vis (CHCl_3_): *λ*_max_ (*ϵ*)=546 (78 874 mol^−1^ dm^3^ cm^−1^); fluorescence (CHCl_3_): *λ*_ex_=495 nm; *λ*_em_=556 nm; HRMS (MALDI‐TOF): *m/z* calcd for C_54_H_70_N_2_O_4_Ge: 884.4562 [*M*]; found: 884.4503; FTIR (cm^−1^): *ṽ*=2954, 2926 and 2856 (aliphatic C−H), 1693 and 1655 (C=O), 1588 (C=C), 1457 and 1400 (aliphatic C−H), 1297 (C−N).

PDIGe_2_: ^1^H NMR (400 MHz, CDCl_3_): *δ*=8.87–8.75 (d, 4H, aryl *H*), 5.30–5.20 (m, 2H, C*H*), 2.34–2.21 (m, 4H, C*H*_2_), 1.92–1.79 (m, 4H, C*H*_2_), 1.50–1.42 (m, 8H, C*H*_2_), 1.31–1.19 (m, 36H, C*H*_2_), 1.19–1.15 (d, 12H, C*H*_3_), 0.84–0.79 ppm (t, 12H, C*H*_3_). ^13^C NMR (100.6 MHz, CDCl_3_): *δ*=165.91, 164.88 (CO), 146.31, 146.23, 140.10, 136.26, 135.47, 129.98, 122.89, 122.60, 121.88 (aryl *C*), 54.83, 54.74 (*C*H), 32.72, 31.93, 29.44, 27.11_,_ 22.76, 14.19 (*C*H_2_), 9.65, 6.58 ppm (*C*H_3_). UV/Vis (CHCl_3_): *λ*_max_ (*ϵ*)=561 (73 679 mol^−1^ dm^3^ cm^−1^); fluorescence (CHCl_3_): *λ*_ex_=495 nm; *λ*_em_=571 nm; HRMS (MALDI‐TOF): *m/z* calcd for C_58_H_78_N_2_O_4_Ge_2_: 1012.4417 [*M*]; found: 1012.5630; FTIR (cm^−1^): ṽ =2953, 2925, 2870 and 2857 (aliphatic C−H), 1690 and 1651 (C=O), 1586 (C=C), 1457 and 1414 (aliphatic C−H), 1277 (C−N).

Deposition Number 2256332 (for PDIGe_2_) contains the supplementary crystallographic data for this paper. These data are provided free of charge by the joint Cambridge Crystallographic Data Centre and Fachinformationszentrum Karlsruhe Access Structures service.

**Solar cell fabrication and characterization**: Organic solar cells were assembled on commercially available indium tin oxide (ITO) coated glass substrates (15×15 mm, 15 Ω/sq, Luminescence Technology Corp.) with the inverted device structure ITO/ZnO/active layer/MoO_3_/Ag. Prior to the fabrication, the substrates were cleaned with deionized water, acetone and sonication in isopropanol (60 min, 40 °C). The substrates were treated with oxygen plasma (3 min, FEMTO, Diener Electronics) before the ZnO (30 nm) was deposited by spin coating (speed: 4000 rpm, ramp: 2000 rpm s^−1^, time 30 s) and subsequent thermal annealing at 150 °C (15 min) in air. The zinc oxide precursor solution was prepared by dissolving zinc acetate dihydrate (0.5 g, 2.3 mmol) in 2‐methoxyethanol (5 mL) and ethanolamine (150 μL, 2.5 mmol). For the active layer, the respective acceptor was dissolved in chlorobenzene and put to the polymeric donor PBDB‐T or PM6 in a D : A weight ratio of 1 : 1 in a concentration of 10 mg mL^‐1^ of the donor. The mixture was stirred overnight at a temperature of 70 °C. The spin coating speed for depositing the active layer was varied from 1000 to 4000 rpm at 500 to 1000 rpm s^‐1^ and 60 s followed by a drying step with 4000 rpm, 4000 rpm s^‐1^ for 5 s. The hole transport layer MoO_3_ (10 nm) and electrode Ag (100 nm) were deposited on the substrates via thermal evaporation through a shadow mask, giving six cells on each substrate with an active area of 0.09 cm^2^.

**Current‐voltage characteristics** were measured using a Keithley 2400 SourceMeter and a Dedolight DEB400D lamp as light source with an intensity of 100 mW cm^‐2^. A shadow mask was used for the measurements defining the active area to be 0.07 cm^2^.

**Light intensity** dependent measurements were done with the same setup as the measurement of the current‐voltage plots. *J*_SC_ and *V*_OC_ were measured under different intensities of the incoming light using neutral density filters.

**External quantum efficiency (EQE)** measurements were done with a MuLTImode 4‐AT monochromator (Amko) equipped with a xenon lamp (LPS 210‐U, Amko), a lock‐in amplifier (Stanford Research Systems, Model SR830) and a Keithley 2400 SourceMeter. The monochromatic light was chopped at a frequency of 30 Hz and the measurement was performed in nitrogen atmosphere. The calibration was done with a silicon photodiode (818‐UV/DB, Newport Corporation) and the spectra were recorded in the range of 380–900 nm.

**Atomic force microscopy (AFM)** height and phase images were taken on the Tosca 400 from Anton Paar with a Cantilever from NanoWorld with a resonance frequency of 285 kHz and a Force Constant of 42 N m^−1^. All images were recorded in tapping mode.

**Electron and hole mobilities** of the PM6:acceptor active layers were measured using the space charge limited current (SCLC) method. The device structure for the hole only devices was ITO/PEDOT:PSS/active layer/MoO_3_/Ag and for the electron only devices ITO/ZnO/active layer/Al. The JV curves of the devices in the dark were measured in a voltage range between −0.5 and 3 V. The mobilities were calculated from the slope *α* of the *J* ^1/2^ vs. *V* plots (Figure S28). It was determined using the following equation: *μ*=(*α* ^2^8 *L* ^3^)/(9*ϵ*_0_ *ϵ*_r_), where *μ* is the mobility, *α* the slope and L is the thickness of the respective active layer. *ϵ*_0_ represents the vacuum permittivity (8.85 CV^−1^ s^−1^) and *ϵ*_r_ is the relative permittivity (3.0). To determine *α*, the linear range was fitted between 1.5 and 2.5 V for each measurement.

## Supporting Information

A further description of measurement techniques and additional characterization data are included in the Supporting Information, as are additional references.[^23^, ^28^, ^38^]

## Conflict of interest

There are no conflicts to declare.

1

## Supporting information

As a service to our authors and readers, this journal provides supporting information supplied by the authors. Such materials are peer reviewed and may be re‐organized for online delivery, but are not copy‐edited or typeset. Technical support issues arising from supporting information (other than missing files) should be addressed to the authors.

Supporting Information

## Data Availability

The data that support the findings of this study are available from the corresponding author upon reasonable request.
