# Supplementary material for: Silicon‐ and Germanium‐Functionalized Perylene Diimides: Synthesis, Optoelectronic Properties, and Their Application as Non‐fullerene Acceptors in Organic Solar Cells
Source: Chemistry. 2023 Aug 28;29(57):e202301337. doi: 10.1002/chem.202301337 (PMC10946824; doi:10.1002/chem.202301337)
Supplement: Supplementary file 1 — Supporting Information [file CHEM-29-0-s001.pdf]

Chemistry–A European Journal

Supporting Information

Silicon- and Germanium-Functionalized Perylene Diimides: Synthesis, Optoelectronic Properties, and Their Application as Non-fullerene Acceptors in Organic Solar Cells

Bettina Schlemmer, Aileen Sauermoser, Sarah Holler, Elena Zuccalà, Birgit Ehmann, Matiss Reinfelds, Roland C. Fischer, Heinz Amenitsch, Jose M. Marin-Beloqui, Lucie Ludvíková, Tomáš Slanina, Michael Haas,* Thomas Rath,* and Gregor Trimmel

General Information

Reagents and solvents were purchased from Merck, Acros, TCI, abcr, Fluorochem, Sigma Aldrich and Fisher Scientific and used as received. The polymers for solar cell fabrication - PBDB-T (poly[(2,6-(4,8-bis(5-(2-ethylhexyl)thiophen-2-yl)-benzo[1,2-b:4,5-b']dithiophene))-alt-(5,5-(1',3'-di-2-thienyl-5',7'-bis(2-ethylhexyl)benzo[1',2'-c:4',5'-c']dithiophene-4,8-dione)], CAS: 1415929-80-4), PM6/PBDB-T-2F (Poly[(2,6-(4,8-bis(5-(2-ethylhexyl-3-fluoro)thiophen-2-yl)-benzo[1,2-b:4,5-b']dithiophene))-alt-(5,5-(1',3'-di-2-thienyl-5',7'-bis(2-ethylhexyl)benzo[1',2'-c:4',5'-c']dithiophene-4,8-dione)], CAS: 1802013-83-7), were purchased from 1-material. Thin layer chromatography was done on TLC Silica gel 60 F_{254} (aluminum sheets, Merck). Column chromatography was done using self-filled columns from Biotage (silica gel 0.04-0.063 mm) and packed columns from Macherey Nagel (CHROMABOND Flash, RS 80, silica gel 40-63 µm) using the "Selekt" flash chromatography instrument (Biotage), as well as on self-packed columns (silica gel 0.04-0.063 mm).

Material Synthesis

5,6,12,13-di(diethylsilyl)-2,9-di(tridecan-7-yl)anthra[2,1,9-def:6,5,10-d'e'f']diisoquinoline-1,3,8,10(2H,9H)-tetraone (PDSi) and 5,6,-diethylsilyl-2,9-di(tridecan-7-yl)anthra[2,1,9-def:6,5,10-d'e'f']diisoquinoline-1,3,8,10(2H,9H)-tetraone ($PDSi_2$)

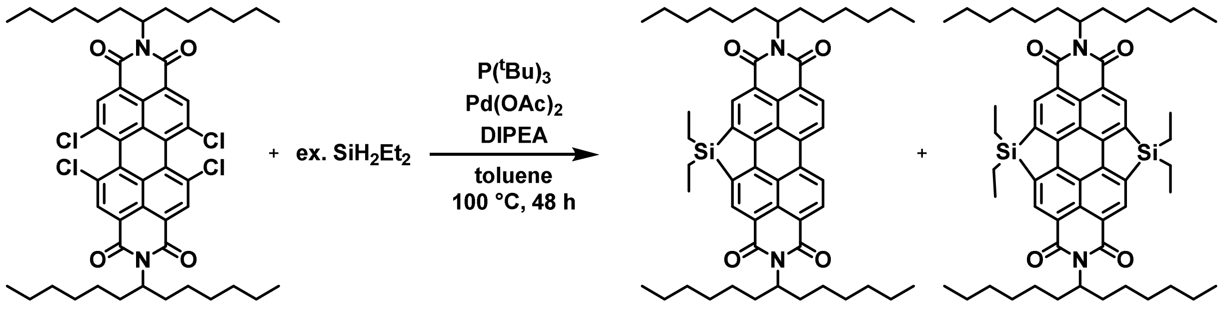

PDICl4 PDISi PDISi2

Figure S1. Synthesis scheme of the silicon containing non-fullerene acceptors PDISi and $PDISi_2$.

5,6,12,13-di(diethylgermyl)-2,9-di(tridecan-7-yl)anthra[2,1,9-def:6,5,10-d'e'f']diisoquinoline-1,3,8,10(2H,9H)-tetraone (PDIGe) and 5,6,-diethylgermyl-2,9-di(tridecan-7-yl)anthra[2,1,9-def:6,5,10-d'e'f']diisoquinoline-1,3,8,10(2H,9H)-tetraone ($PDIGe_2$)

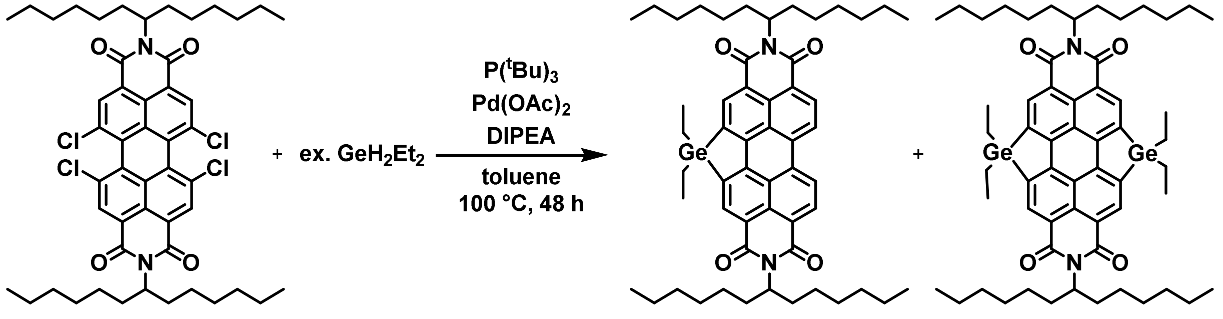

PDICl4 PDIGe PDIGe2

Figure S2. Synthesis scheme of the silicon containing non-fullerene acceptors PDIGe and $PDIGe_2$.

Structural Analysis

NMR spectra

^{1}H (399.95 MHz, 299.95 MHz or 199.97 MHz), ^{13}C (100.58 MHz or 75.43 MHz) and ^{29}Si (39.73 MHz) NMR spectra were recorded on either a JNM-ECZ400 JEOL 400 MHz system with auto sampler and Royal HFX probe head, a Bruker AVANCE III 300 MHz spectrometer with an auto sampler or a Bruker AVANCE DPX 200 MHz spectrometer in either CD_2Cl_2 or $CDCl_3$ solution (99.5 atom %, D) using the internal ^{2}H-lock signal of the solvent.

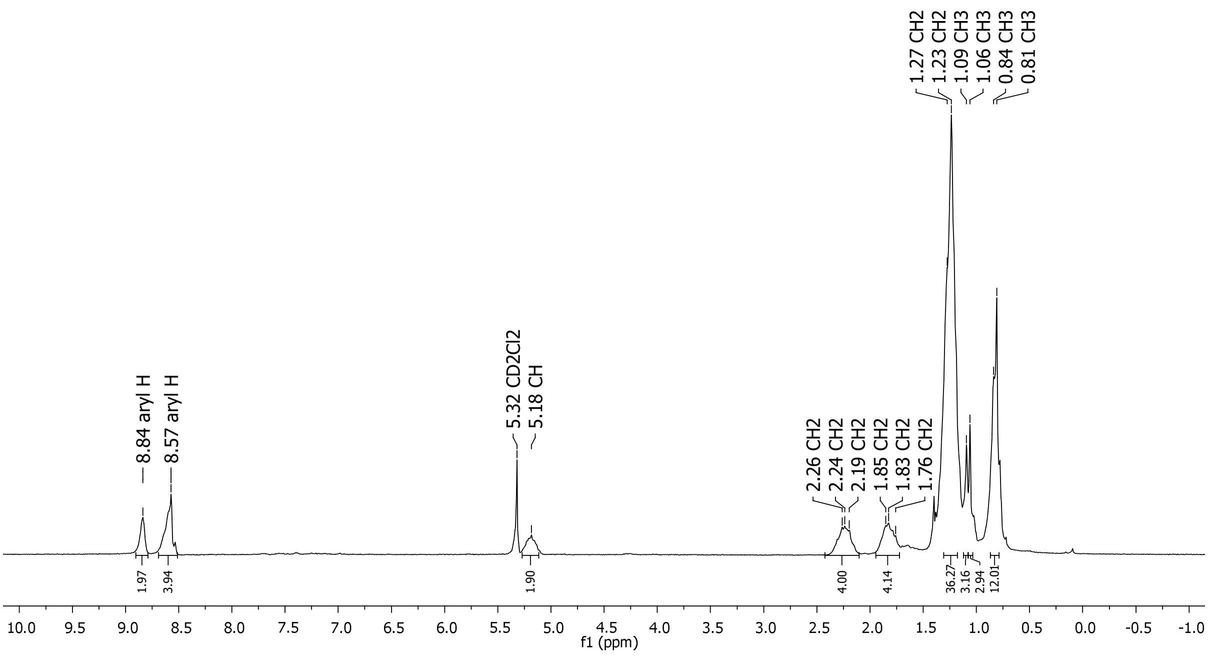

Figure S3. ^{1}H-NMR spectrum of PDISi (CD_2Cl_2 solution, ppm, 200 MHz).

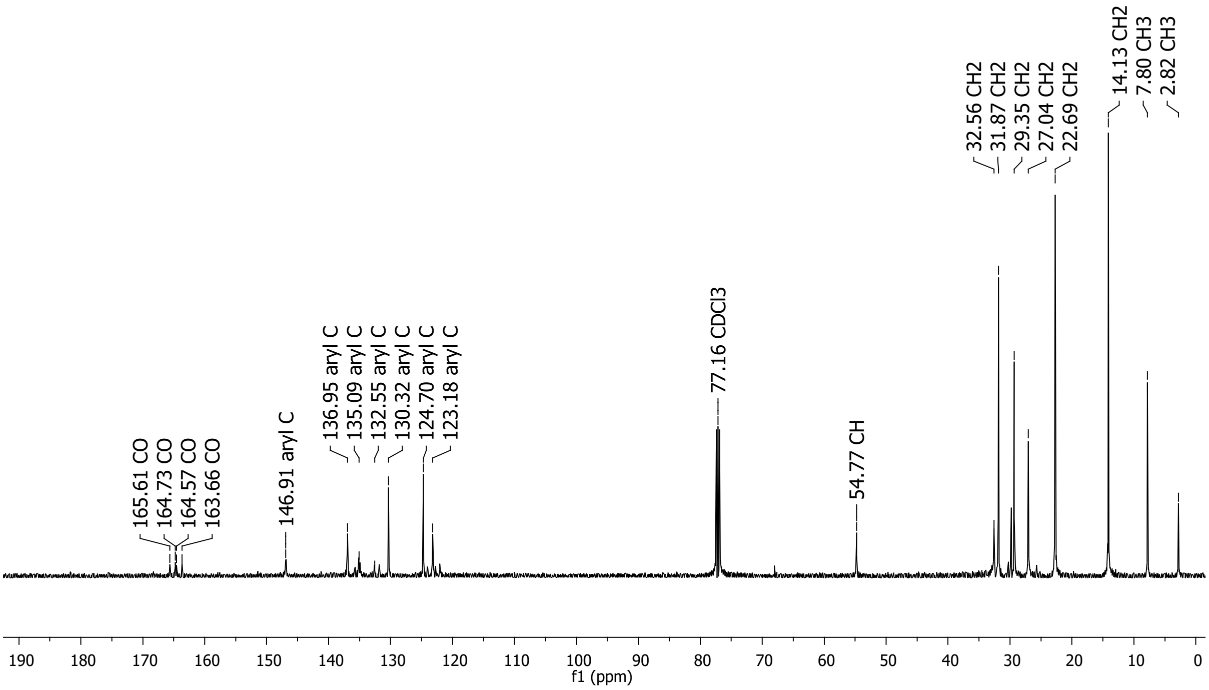

Figure S4. ^{13}C-NMR spectrum of PDISi ($CDCl_3$ solution, ppm, 76 MHz).

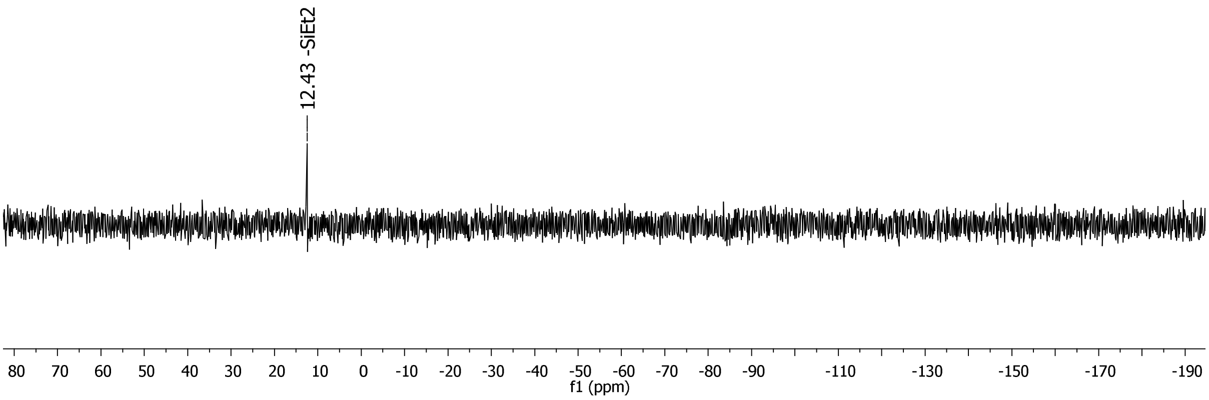

Figure S5. ^{29}Si-NMR spectrum of PDISi ($CDCl_3$ solution, ppm, 40 MHz).

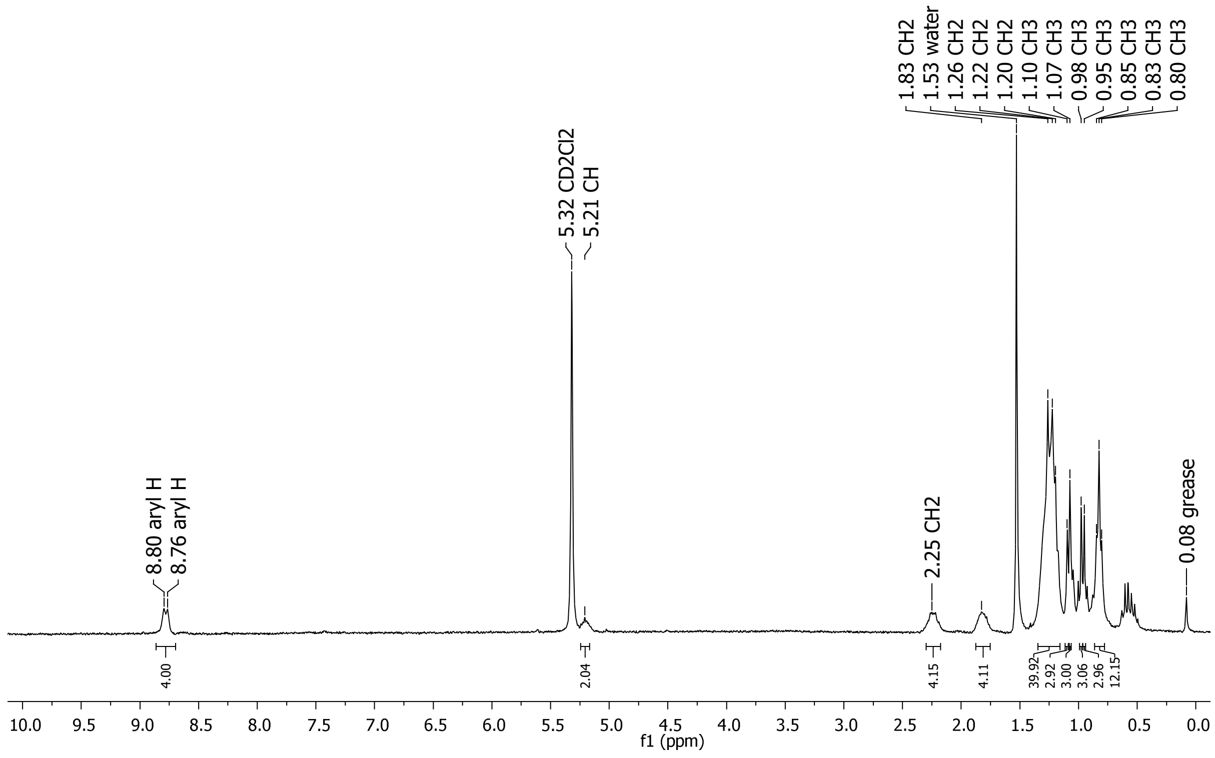

Figure S6. ^{1}H-NMR spectrum of $PDISi_2$ (CD_2Cl_2 solution, ppm, 200 MHz).

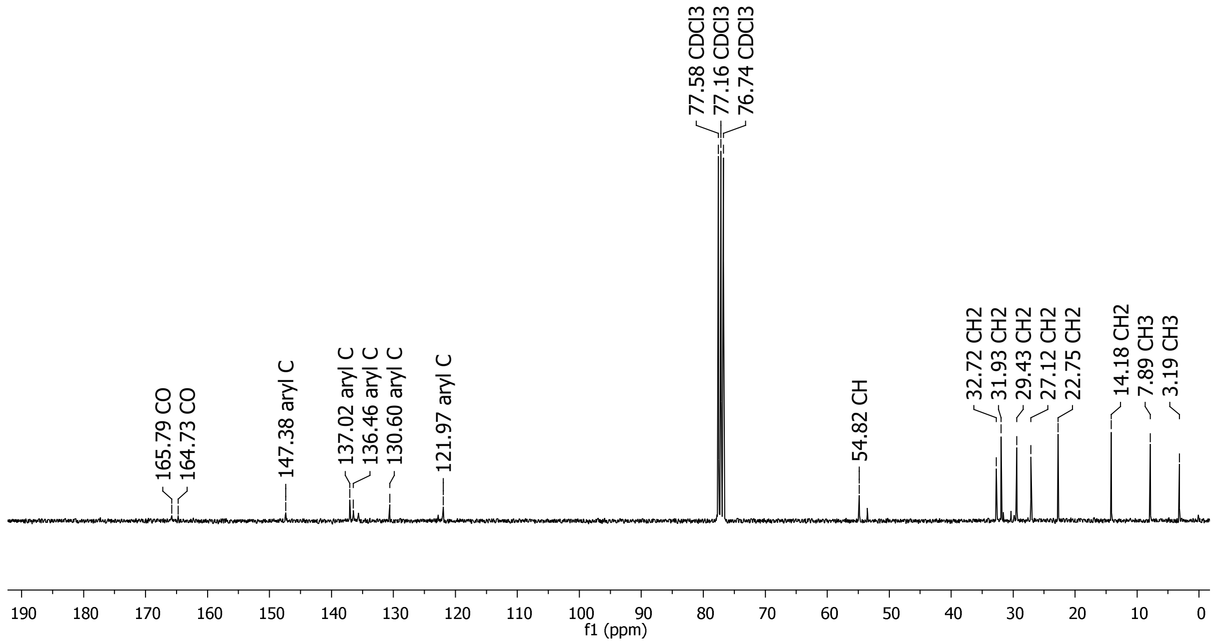

Figure S7. ^{13}C-NMR spectrum of $PDISi_2$ ($CDCl_3$ solution, ppm, 76 MHz).

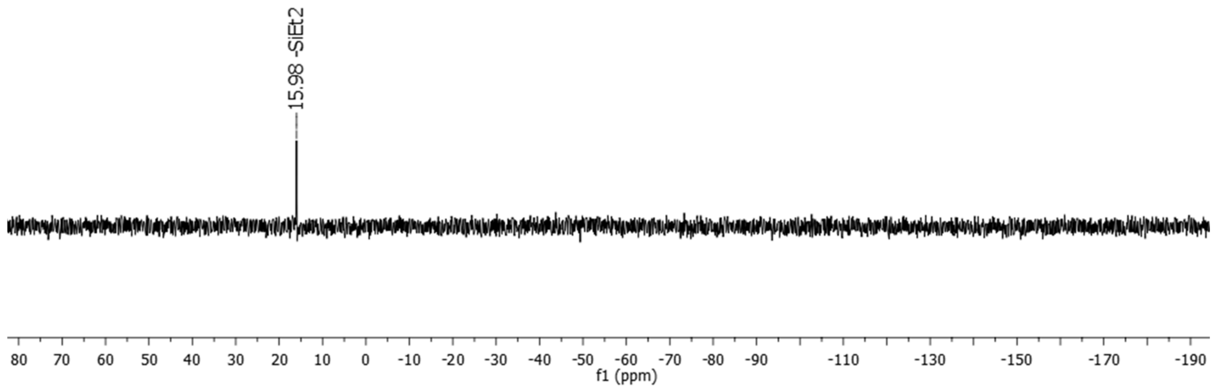

Figure S8. ^{29}Si-NMR spectrum of $PDISi_2$ ($CDCl_3$ solution, ppm, 40 MHz).

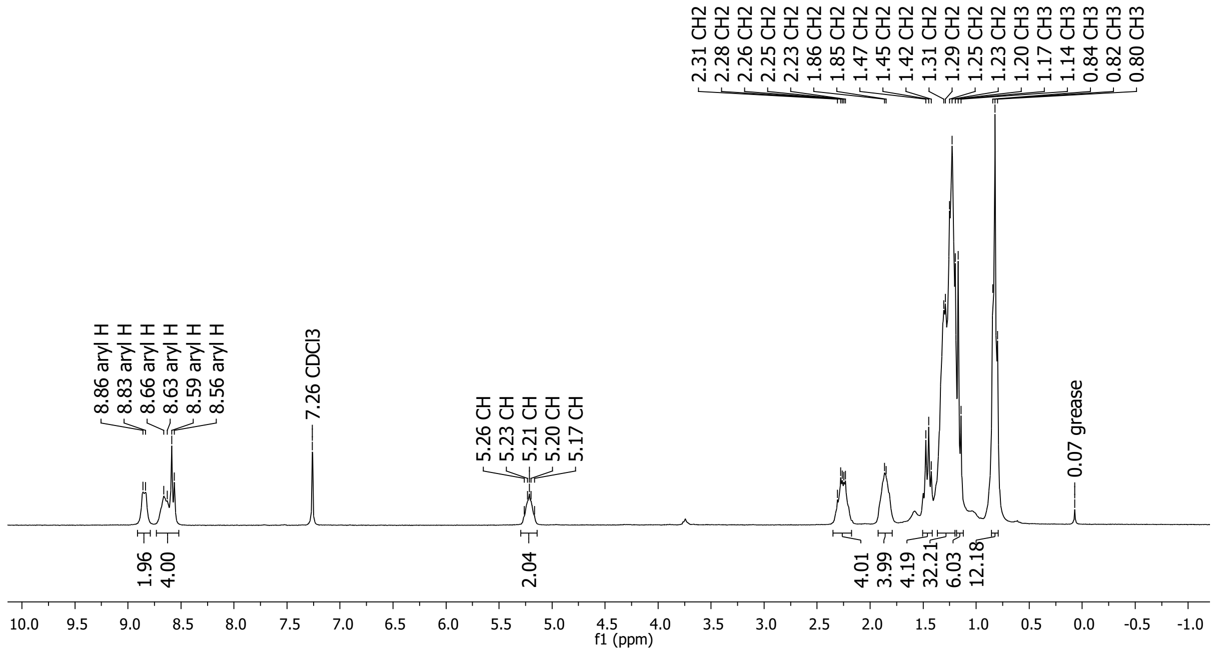

Figure S9. ^{1}H-NMR spectrum of PDIGe ($CDCl_3$ solution, ppm, 300 MHz).

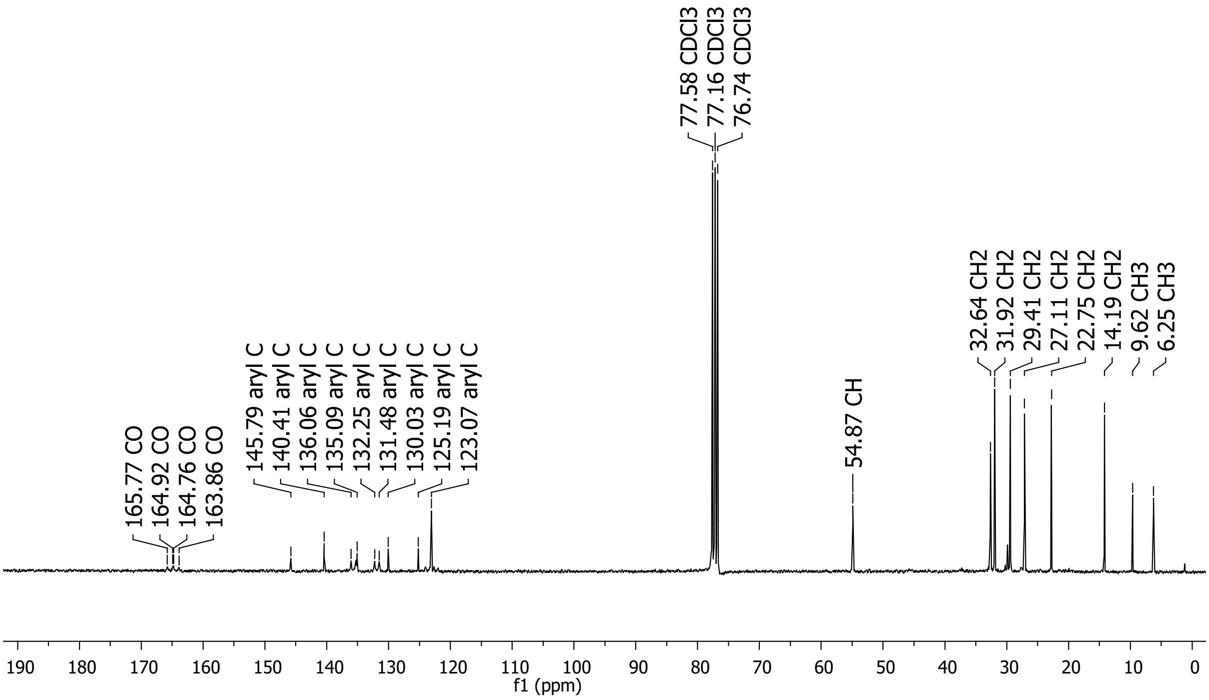

Figure S10. ^{13}C-NMR spectrum of PDIGe ($CDCl_3$ solution, ppm, 100.6 MHz).

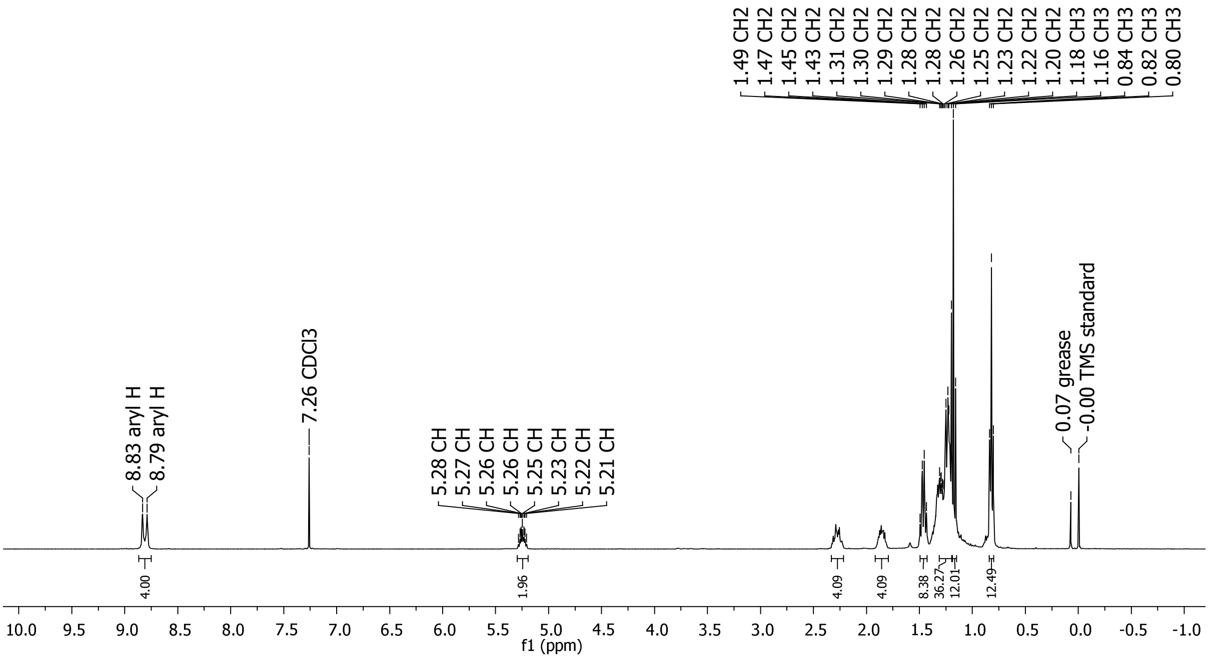

Figure S11. ^{1}H-NMR spectrum of $PDIGe_2$ ($CDCl_3$ solution, ppm, 400 MHz).

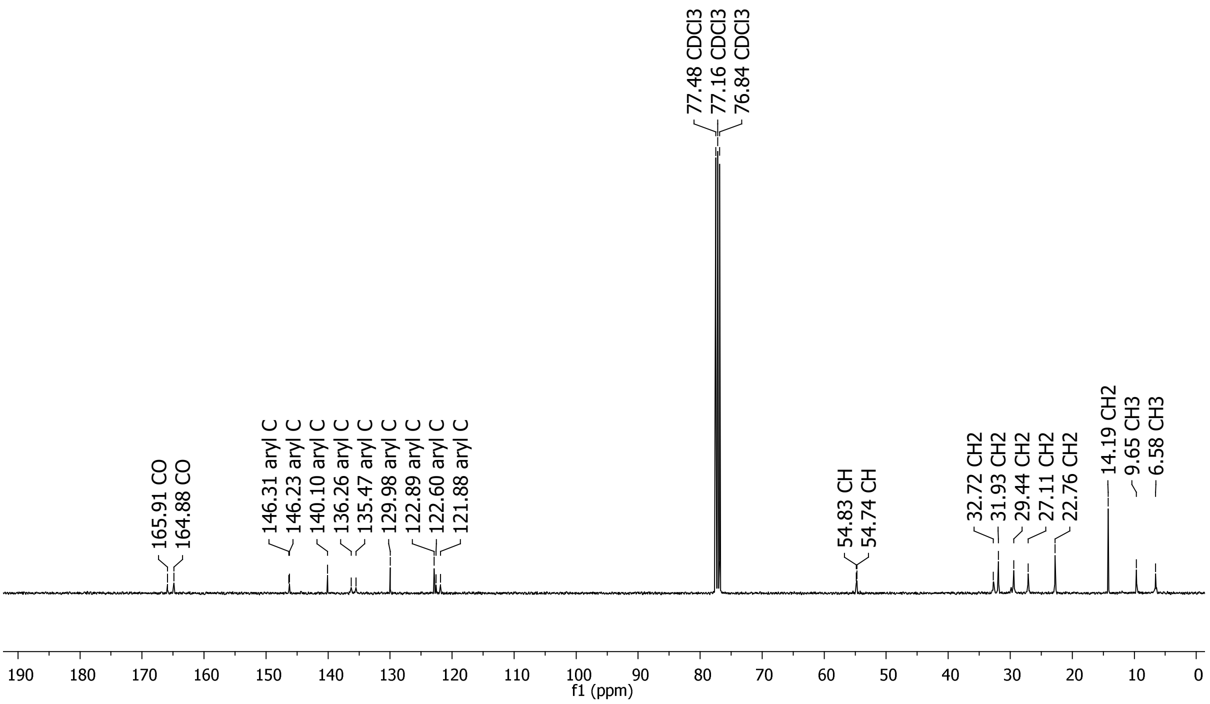

Figure S12. ^{13}C-NMR spectrum of $PDIGe_2$ ($CDCl_3$ solution, ppm, 100.6 MHz).

IR Spectra

Infrared spectroscopy was done on a Bruker Alpha spectrometer in transmission mode. The molecules were deposited from chlorobenzene solutions via drop coating on undoped Si wafer substrates.

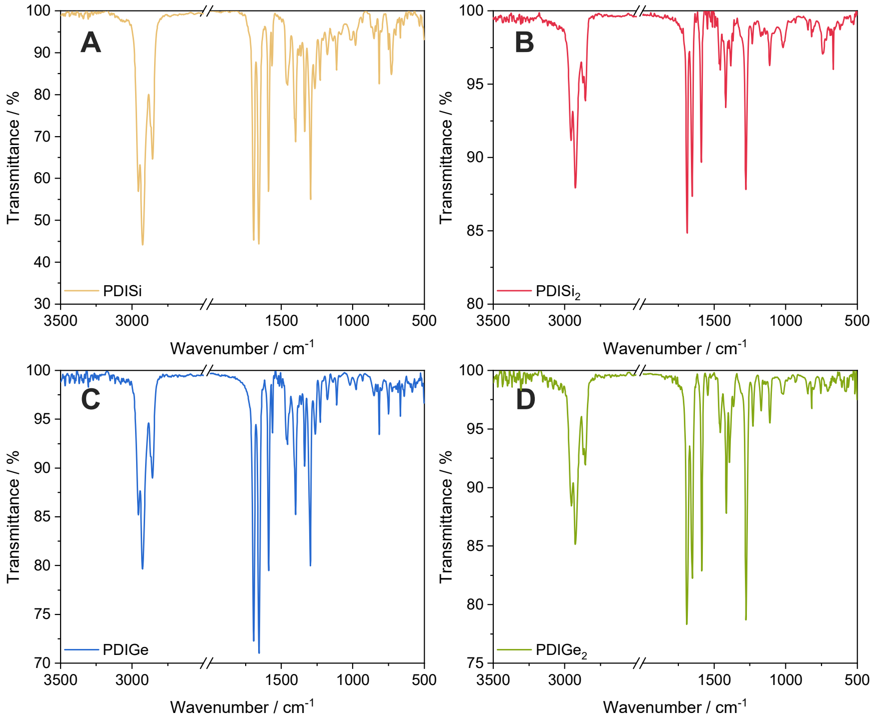

Figure S13. Infrared spectra of A) PDISi, B) $PDISi_2$, C) PDIGe and D) $PDIGe_2$ in thin films.

Mass Spectrometry

The mass spectra were recorded on the 'Micromass MALDI micro MX' mass spectrometer from Waters. The matrix was trans-2-[3-(4-tert-butylphenyl)-2-methyl-2-propenylidene]malononitrile (DCTB) in a concentration of 10 mg mL^{-1} in THF; the sample had a concentration of 1 mg mL^{-1} in DCM in a mixing ratio matrix/sample=7/2. The positive mode was used, producing M^+ molecule ions. The reference material used was polyethylene glycol (PEG). The MS measurements were performed by Karin Bartl and the data analysis was done with the MassLynx V4.1 software.

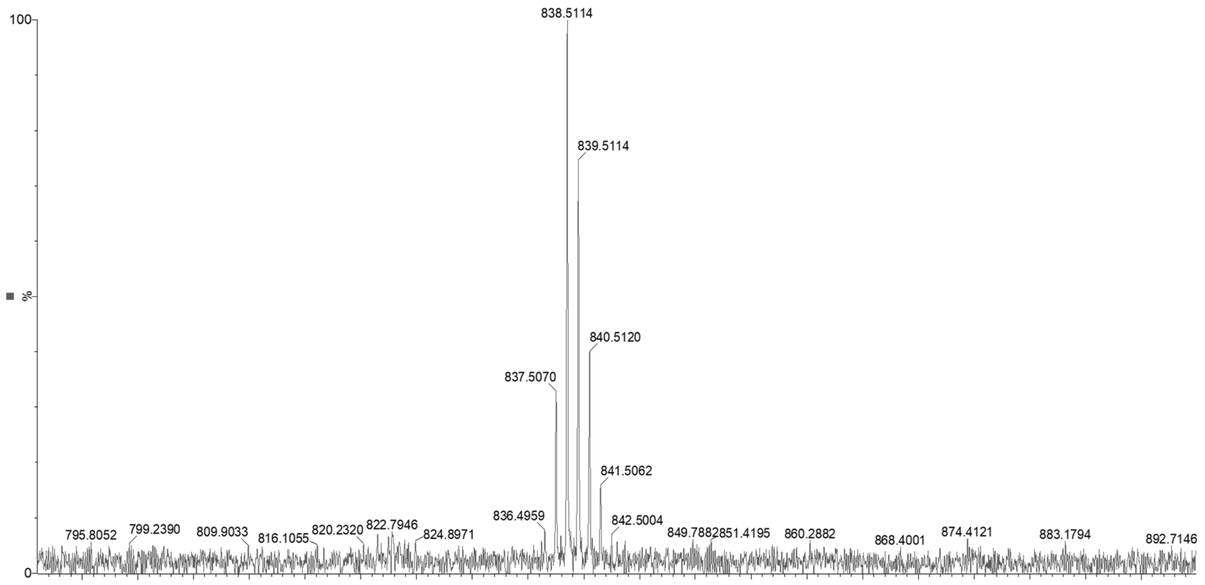

Figure S14. Mass spectrum of PDISi.

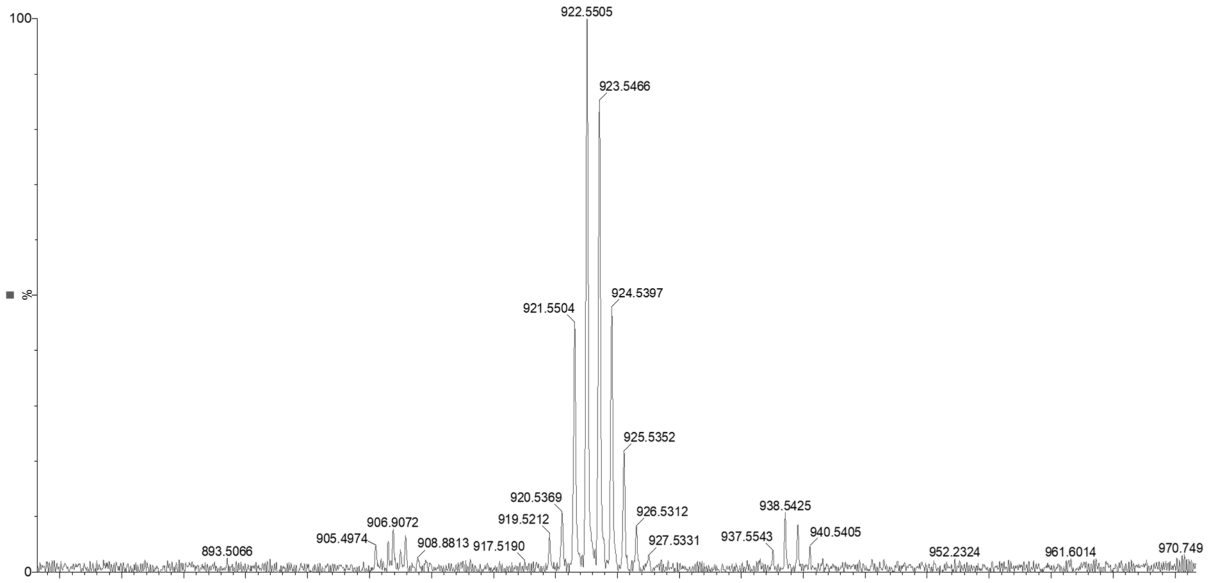

Figure S15. Mass spectrum of $PDISi_2$.

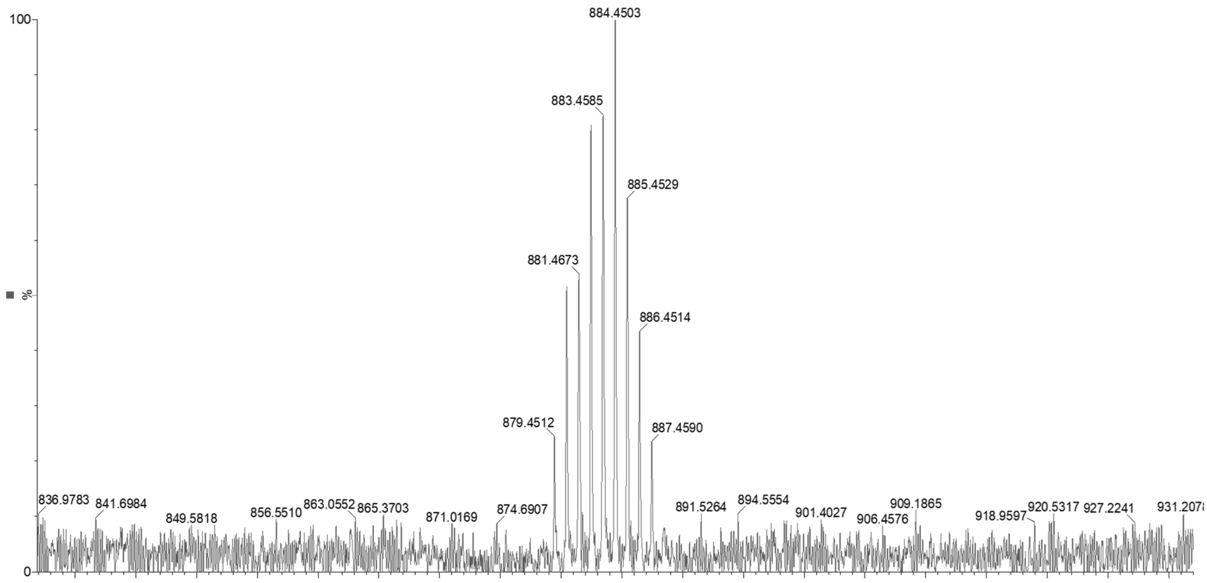

Figure S16. Mass spectrum of PDIGe.

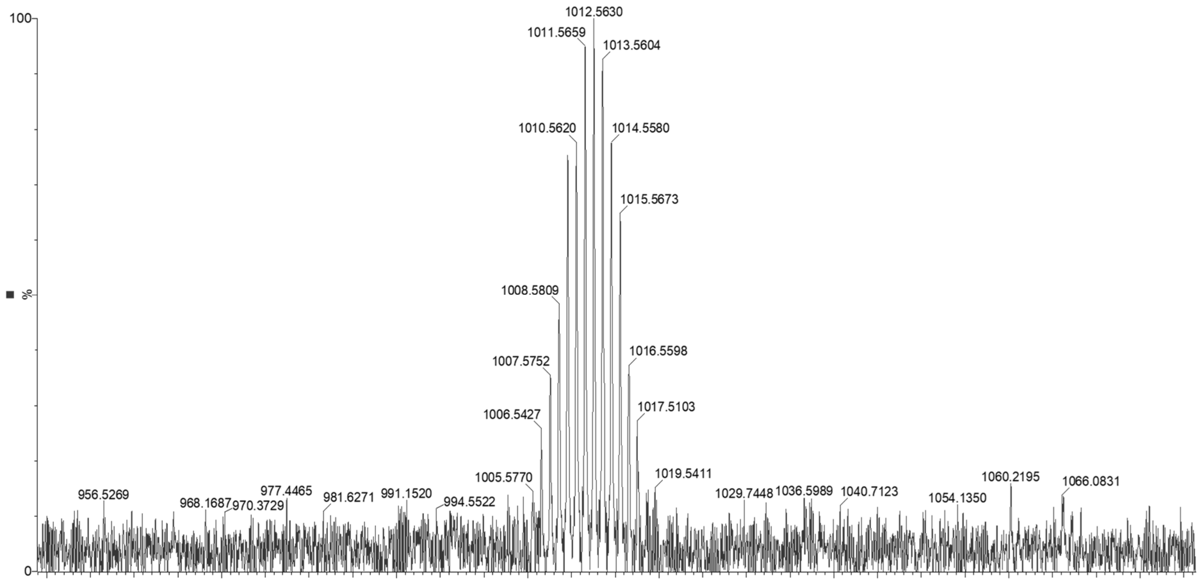

Figure S17. Mass spectrum of $PDIGe_2$.

Single-crystal X-ray Diffraction

The crystal suitable for single-crystal X-ray diffractometry was removed from a vial and covered with a layer of silicone oil. A single crystal was selected, mounted on a glass rod on a copper pin, and placed in a cold N_2 stream. XRD data collections for $PDIGe_2$ was performed on a Bruker APEX II diffractometer with use of an Incoatec microfocus sealed tube of Mo Kα radiation (λ=0.71073 Å) and a CCD area detector. Empirical absorption corrections were applied using SADABS or TWINABS.[2] The structures were solved with either the use of direct methods or the intrinsic phasing option in SHELXT and refined by the full-matrix least-squares procedures in SHELXL.[3] or Olex2.[4] The space group assignments and structural solutions were evaluated using PLATON.[5] Non-hydrogen atoms were refined anisotropically. Hydrogen atoms were either located in a difference map or in calculated positions corresponding to standard bond lengths and angles. **Table S1** contains crystallographic data and details of measurements and refinement for $PDIGe_2$. Crystallographic data (excluding structure factors) have been deposited with the Cambridge Crystallographic Data Centre (CCDC) under the following number: $PDIGe_2$: 2256332.

Table S1. Crystallographic data of $PDIGe_2$.

**Compound**	**$PDIGe_2$ (2256332)**
Formula	$C_{58}H_{78}Ge_2N_2O_4$
M_r (g mol^{-1})	1012.40
a (Å)	12.1720(7)
b (Å)	23.3564(14)
c (Å)	19.3274(10)
α (°)	90
β (°)	105.699(3)
γ (°)	90
V (Å3)	5289.7(5)
Z	4
Crystal size (mm)	0.17× 0.06 × 0.05
Crystal system	Monoclinic
Space group	$P2_1/n$
d_{calc} (Mg m^{-3})	1.271
μ (mm^{-1})	1.183
T (K)	100.01
2θ range (°)	2.798 to 51.998
F (000)	2144.0
R_{int}	0.0454
No. of measured, and independent [I > 2σ(I)] reflections	96322, 10393
No. of parameters, restraints	369,723
Δ›max, Δ›min (e Å^{-3})	0.74/-0.73
R1, wR2 (all data)	R1=0.0810 wR2=0.1430
R1, wR2 (>2σ)	R1=0.0610 wR2=0.1317

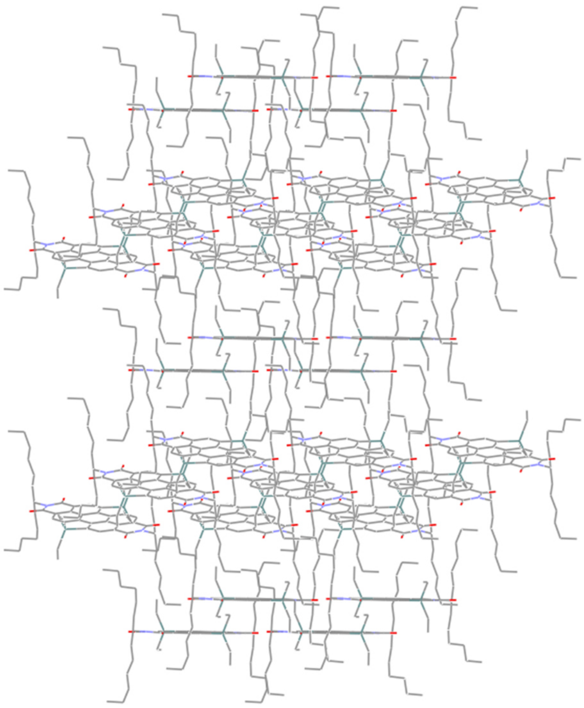

Figure S18. 3D packing of $PDIGe_2$ in the single crystal.

Grazing Incidence Wide Angle X-ray Scattering (GIWAXS)

Samples for the measurements were prepared via drop coating from chlorobenzene solutions on silicon substrates, for some followed by an annealing step of 10 min at 140 °C in inert atmosphere. The measurements were carried out at the Austrian SAXS Beamline 5.2L of the electron storage ring ELETTRA Trieste at a photon energy of 8 keV.[6] For the detection of the GIWAXS images, a Dectris Pilatus3 1M detector was used set to a sample detector distance of 294 mm. The angular calibration of the detector was carried out using silver behenate powder (d-spacing: 58.38 Å). All measurements have been performed with a grazing angle of 1.1°. The scattering of a blank silicon wafer has been subtracted as background from the GIWAXS images. The in-plane line cuts taken at the Yoneda wing (q_r) and out-of-plane line cuts (q_z) along the Ewald sphere (see red boxes in Figure S14A) have been determined with the automatic data processing software SAXSDOG.[7] The images and data were processed using FIT2D and IGOR Pro 7 (Wavemetrics).

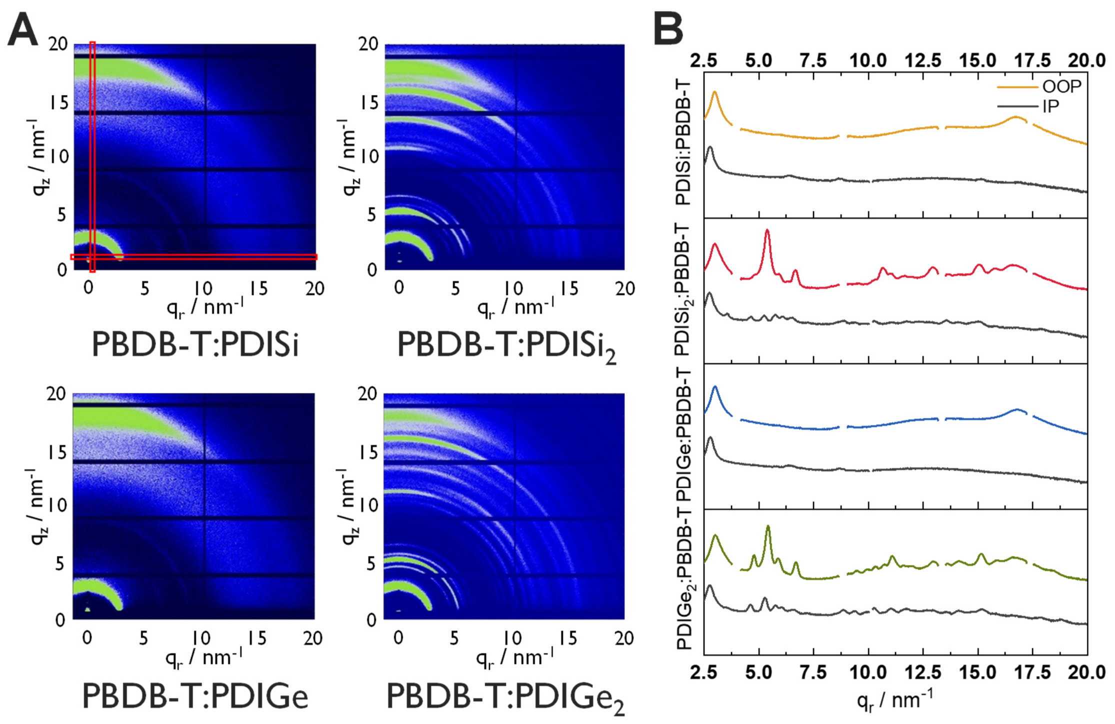

Figure S19. A) 2D GIWAXS images of PBDB-T:PDISi, PBDB-T:PDISi2, PBDB-T:PDIGe and PBDB-T:PDIGe2. B) Line cuts in plane (grey) and out of plane (colored) of PBDB-T:PDISi, PBDB-T:PDISi2, PBDB-T:PDIGe and PBDB-T:PDIGe2. The red boxes indicate the areas used for vertical and horizontal integration.

Optical Analysis

Optical Microscopy

Images were taken with the digital light microscope DSX1000 from Olympus in a magnification of 560x in bright and dark field modes.

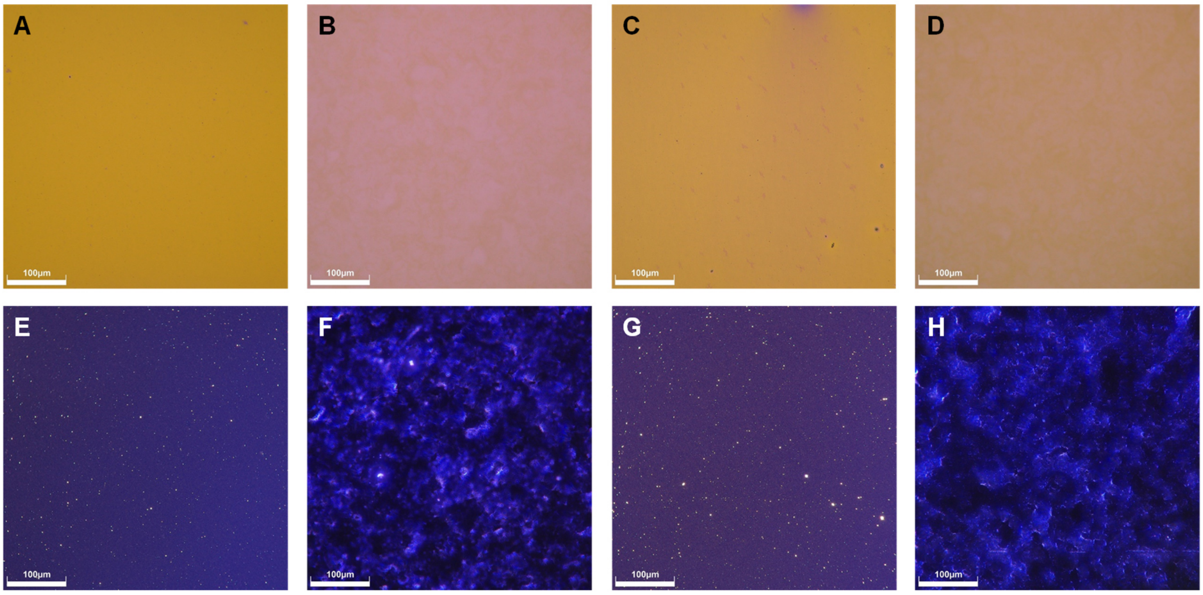

Figure S20. Bright field and dark field light microscope images of A, E) PM6:PDISi B, F) PM6:PDISi$_2$ C, G) PM6:PDIGe and D, H) PM6:PDIGe$_2$ active layers, respectively. It should be noted that the color differences in the microscope images are not the actual color impression of the samples.

Absorption Spectroscopy

Absorption measurements in chloroform solutions and in thin films were done using the Shimadzu spectrophotometer UV-1800 (UV-Vis). A wavelength range of 400 – 700 nm was used.

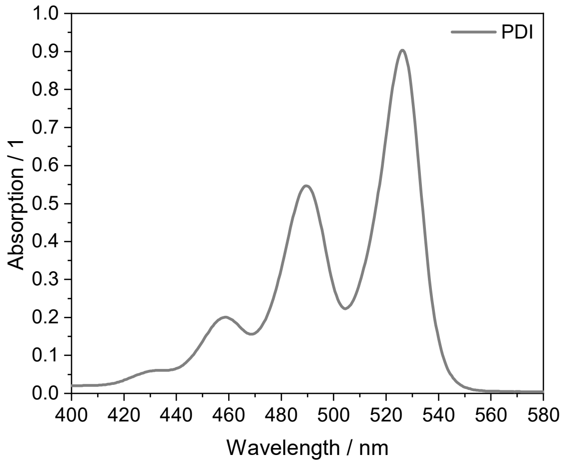

Figure S21. Absorption spectrum of the unsubsituted PDI in $CHCl_3$ solution.

Fluorescence Spectroscopy

Fluorescence (excitation and emission spectra) measurements were done using the F-7000 FL fluorescence spectrophotometer (Hitachi). Luminescence lifetimes were determined using time-correlated single 3 photon counting on a FluoroLog 3 spectrofluorometer (Horiba Scientific) equipped with an NIR-sensitive R2658 photomultiplier (Hamamatsu) and a DeltaHub module controlling a NanoLED (456 nm) laser diode. Lifetimes were calculated using the monoexponential decay model in OriginPro 2021. The relative quantum yields were determined on the same spectrofluorometer and were calculated against the standard 'Fluoreszenzorange' from Kremer Pigmente (perylene diimide with Φ_{fl}=0.95).

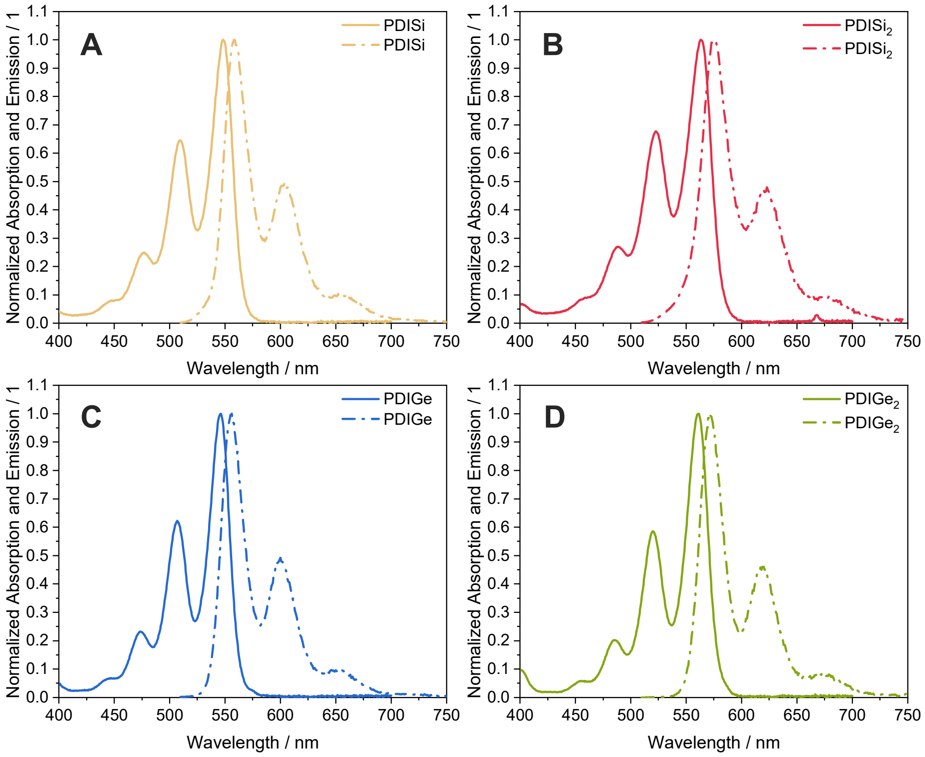

Figure S22. Absorption and fluorescence spectra of A) PDISi, B) $PDISi_2$, C) PDIGe and D) $PDIGe_2$ in $CHCl_3$ solution.

Computational Methods

The computations were performed in the gas phase with the Gaussian 16 revision C.01 program package at the B3LYP/6-31 d,p level of theory with the empirical dispersion correction GD3BJ.[8]

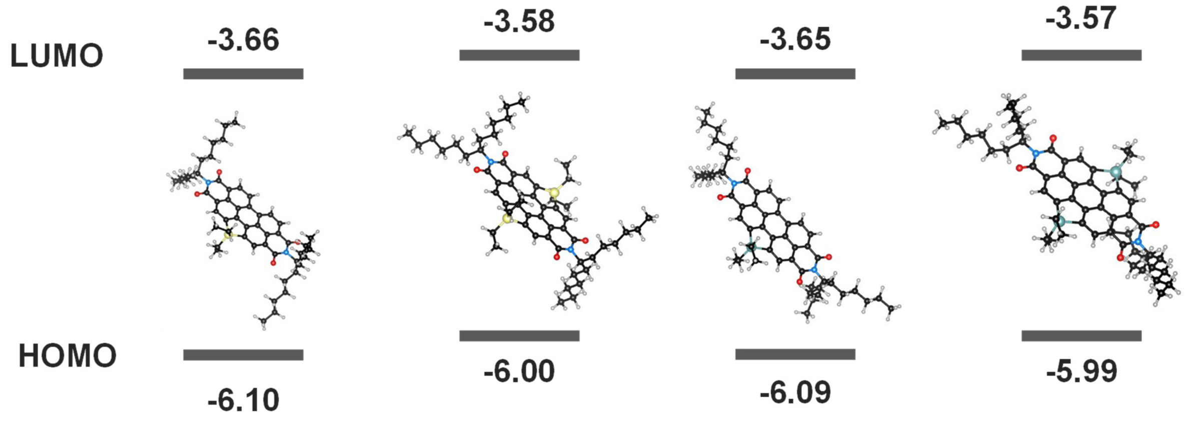

Figure S23. Calculated molecular geometry of all acceptors and their calculated HOMO and LUMO energy levels (from left to right: PDISi, $PDISi_2$, PDIGe, $PDIGe_2$).

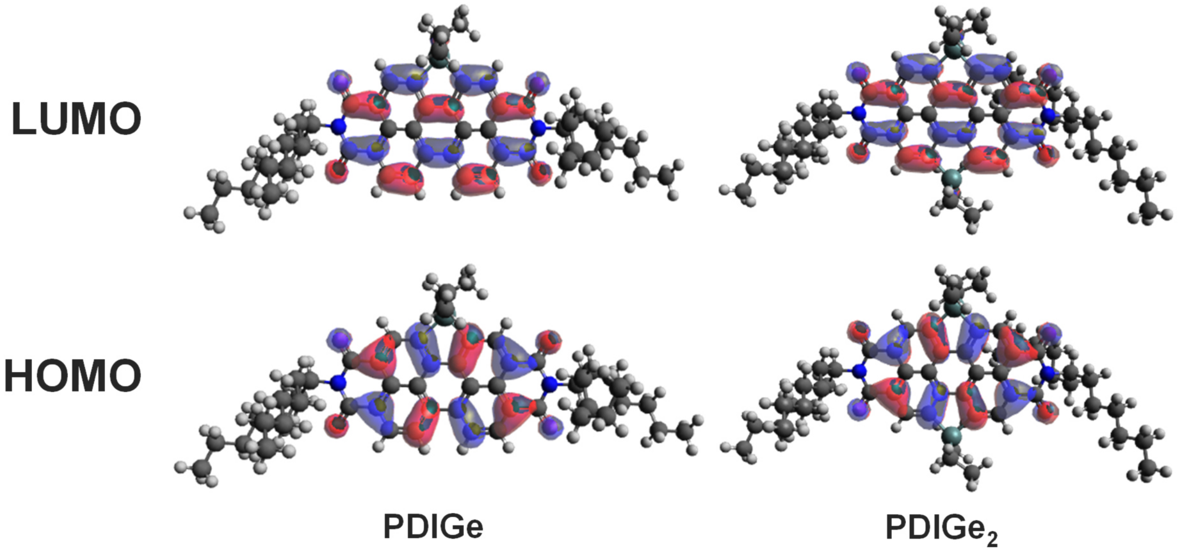

Figure S24. Calculated electron density distribution of HOMOs and LUMOs of PDIGe and $PDIGe_2$.

Cyclic Voltammetry (CV)

CV measurements were performed using a SP 50 single channel potentiostat from BioLogic and the corresponding software EC Lab® (V11.31). A three-electrode setup was used with a Pt-disc working electrode (d=2 mm), a Pt-wire counter electrode (d=0.5 mm) and a non-aqueous Ag/$AgNO_3$ reference electrode (0.5 mm Ag wire in a 0.1 M $AgNO_3$ solution in MeCN). The materials were drop casted on the working electrode and measured using a 0.1 M tetrabutylammoniumhexafluorophosphate ($TBAPF_6$) in acetonitrile (MeCN) as the electrolyte solution (in a nitrogen filled glovebox, with scan speed of 50 mV s^{-1}). The measurements were performed by starting from zero to positive voltages and for each measurement a freshly casted film was used. Calibration was done using the Fc/Fc^+ redox couple as external standard. The HOMO and LUMO energy levels were calculated using the following equations.

$$E_{HOMO} = -(E^{ox}_{onset\ vs.\frac{Fc}{Fc^+}} + 5.39)\ eV \qquad \textbf{Eq. 1}$$

$$E_{LUMO} = E_{HOMO} + E_g^{opt} \qquad \textbf{Eq. 2}$$

The Fermi energy level of NHE vs. vacuum was taken as 5.39 eV, whereas the redox potential of Fc/Fc^+ vs. NHE was taken as 0.64 V. [9], [10]

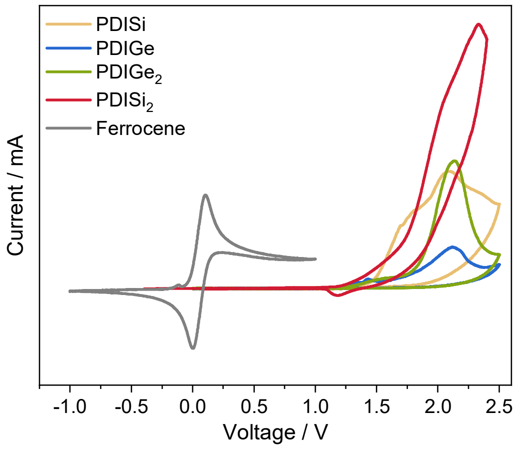

Figure S25. Cyclic voltammograms of PDISi, $PDISI_2$, PDIGe, $PDIGe_2$ and the ferrocene reference.

Organic Solar Cells

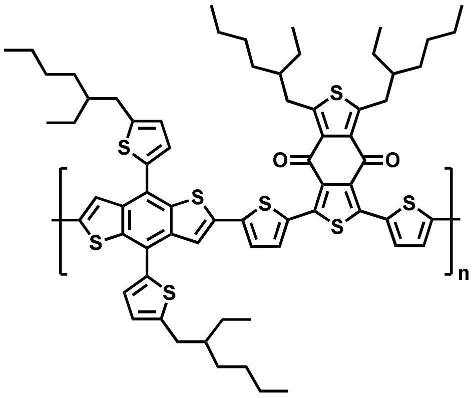

PBDB-T

Figure S26. Structure of the used donor polymer PBDB-T.

PBDB-T based Solar Cells

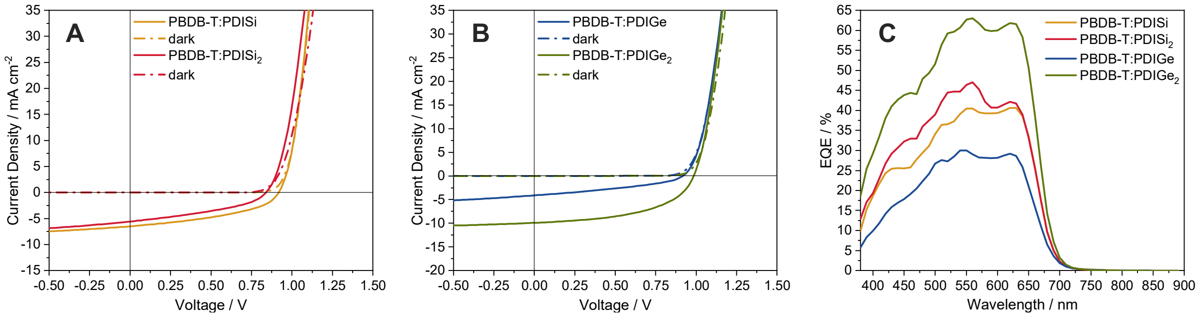

Figure S27. A) JV characteristics of solar cells containing PBDB-T:PDISi and PBDB-T:PDISi2 absorber layers. B) JV characteristics of solar cells containing PBDB-T:PDIGe and PBDB-T:PDIGe2 absorber layers. C) External quantum efficiency (EQE) spectra of the solar cells based on the acceptors combined with PBDB-T.

Table S2. Characteristic parameters of the solar cells with the silicon- and germanium-based non-fullerene acceptors in combination with the donor polymer PBDB-T; all solar cells have an active layer thickness of 60 nm and were annealed at 140 °C; mean values and standard deviations are calculated from 10 solar cells (best values in brackets).

Active Layer	V_{OC} [V]	I_{SC} [mA cm^{-2}]	FF [%]	PCE [%]	α / n
PBDB-T:PDISi	0.91 ± 0.01 (0.91)	6.11 ± 0.28 (6.48)	41.9 ± 0.99 (43.1)	2.32 ± 0.15 (2.53)	0.97 / 1.27
PBDB-T:PDISi2	0.83 ± 0.01 (0.83)	4.96 ± 0.44 (5.56)	38.3 ± 0.65 (39.0)	1.56 ± 0.15 (1.78)	0.90 / 1.13
PBDB-T:PDIGe	0.88 ± 0.01 (0.91)	3.76 ± 0.07 (4.10)	35.9 ± 0.37 (36.5)	1.19 ± 0.02 (1.36)	0.92 / 1.39
PBDB-T: PDIGe2	0.97 ± 0.01 (0.97)	9.18 ± 0.69 (9.92)	50.7 ± 1.16 (51.8)	4.48 ± 0.34 (4.95)	0.99 / 1.18

Electron and Hole Mobilities

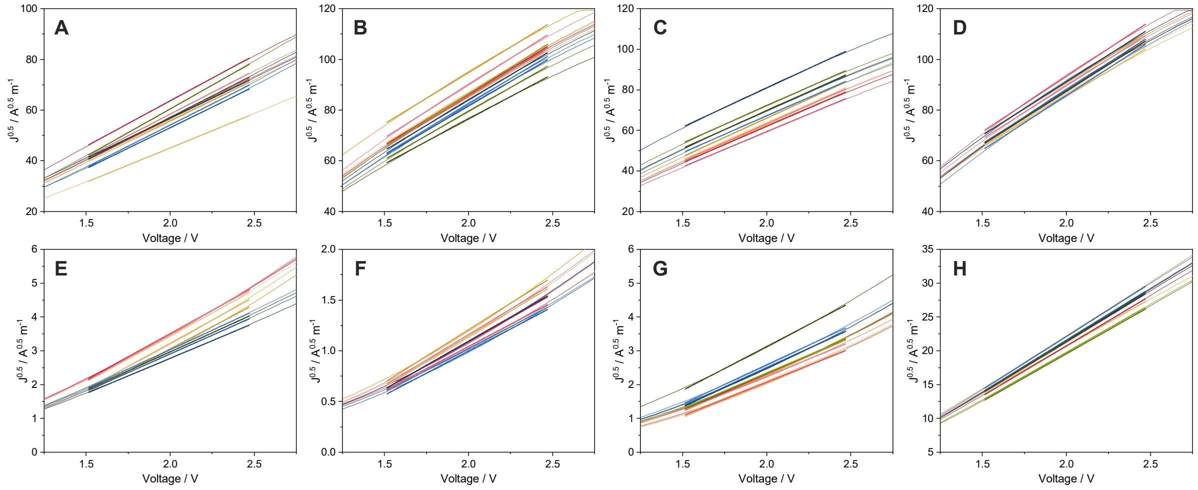

Figure S28. $J^{0.5}$ vs. voltage plots of the hole (top) and electron (bottom) only devices in the following order PM6:PDISi (A, E), PM6:PDISi$_2$ (B, F), PM6:PDIGe (C, G) and PM6:PDIGe$_2$ (D, H).

Transient Absorption Spectroscopy

Transient absorption experiments were carried out in transmission mode using a commercially available apparatus from Ultrafast Systems. Briefly, an Ytterbium femtosecond laser was used to generate 1030 nm with 400 mJ pulses at 1 kHz. The output was split into two parts, where 75% of the 1030-nm pulses were used to pump a collinear Optical Parametric Amplifier (OPA, APOLLO-Y) tuned to pump 550 nm pulses (ca. 250 fs, 80 nJ at the sample position) for sample excitation and 25% were used for generation of supercontinuum white light probe pulses by focusing into a crystal giving a probe spectrum ranging from 500 - 950 nm. Probe pulses were delayed via an optical motorized delay line with 2 ns time window. The pump and probe pulses were focused colinearly into the sample to spot sizes of ca. 250 and 50 µm full width at half maximum, respectively. The isotropic spectral signals were secured by use of an achromatic broadband depolarizer for the pump (ThorLabs, DPP-25-A). The data were corrected for chirp. The sample was randomly moved at 1 mm/s speed through the measurement. The stability of the sample was verified by recording steady-state absorption spectra before and after each measurement.

The films for the TAS measurements were spin coated on glass substrates (15x15 mm, film thickness: approx. 60 nm), annealed and encapsulated with a second glass slide and epoxy glue.

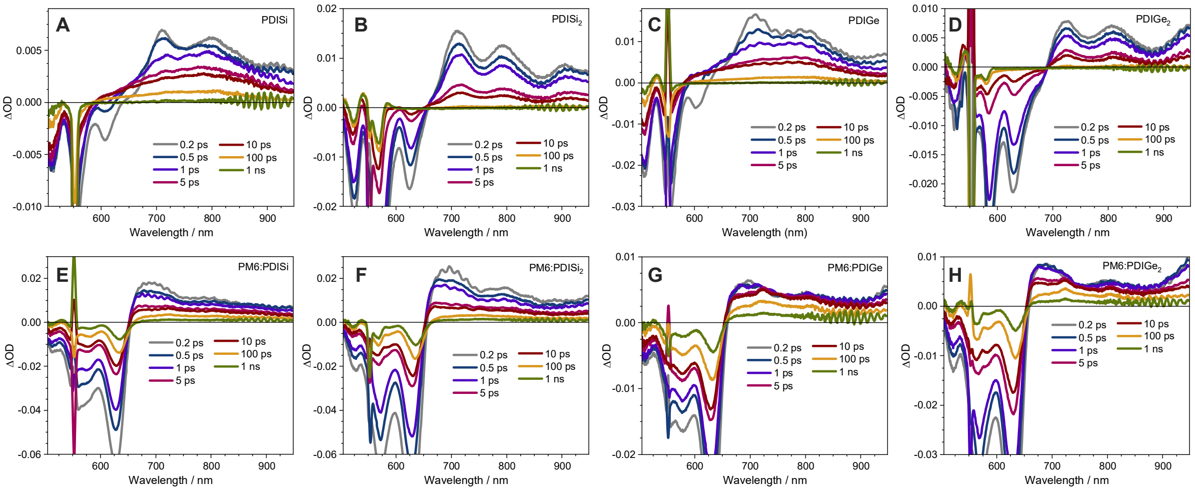

Figure S29. Transient absorption spectra at several delay times of thin films of the neat acceptors A) PDISi, B) $PDISi_2$, C) PDIGe and D) $PDIGe_2$ and the blend films E) PM6:PDISi, F) PM6:$PDISi_2$, G) PM6:PDIGe and H) PM6:$PDIGe_2$. The samples were excited at 550 nm (E ~ 80 nJ).

References

[1] M. W. Holman, R. Liu, D. M. Adams, *J. Am. Chem. Soc.* **2003**, *125*, 12649.

[2] a) *Bruker APEX2 and SAINT*, Bruker AXS Inc., Madison, Wisconsin, USA, **2012**; b) R. H. Blessing, *Acta Crystallogr. Sect. A* **1995**, *51 (Pt 1)*, 33.

[3] a) G. M. Sheldrick, *Acta Crystallogr. Sect. A* **1990**, *46*, 467; b) G. M. Sheldrick, *Acta Crystallogr. Sect. A* **2008**, *64*, 112; c) G. M. Sheldrick, *Acta Crystallogr. Sect. A* **2015**, *71*, 3.

[4] O. V. Dolomanov, L. J. Bourhis, R. J. Gildea, J. A. K. Howard, H. Puschmann, *J. Appl. Crystallogr.* **2009**, *42*, 339.

[5] a) A. L. Spek, *J. Appl. Crystallogr.* **2003**, *36*, 7; b) A. L. Spek, *Acta Crystallogr. Sect. D* **2009**, *65*, 148.

[6] H. Amenitsch, M. Rappolt, M. Kriechbaum, H. Mio, P. Laggner, S. Bernstorff, *J. Synchrotron Rad.* **1998**, *5*, 506.

[7] M. Burian, C. Meisenbichler, D. Naumenko, H. Amenitsch, *J. Appl. Crystallogr.* **2022**, *55*, 677.

[8] M. J. Frisch, G. W. Trucks, H. B. Schlegel, G. E. Scuseria, M. A. Robb, J. R. Cheeseman, G. Scalmani, V. Barone, B. Mennucci, G. A. Petersson, H. Nakatsuji, M. Caricato, X. Li, H. P. Hratchian, A. F. Izmaylov, J. Bloino, G. Zheng, J. L. Sonnenberg, M. Hada, M. Ehara, K. Toyota, R. Fukuda, J. Hasegawa, M. Ishida, T. Nakajima, Y. Honda, O. Kitao, H. Nakai, T. Vreven, J. A. Montgomery, Jr., J. E. Peralta, F. Ogliaro, M. Bearpark, J. J. Heyd, E. Brothers, K. N. Kudin, V. N. Staroverov, T. Keith, R. Kobayashi, J. Normand, K. Raghavachari, A. Rendell, J. C. Burant, S. S. Iyengar, J. Tomasi, M. Cossi, N. Rega, J. M. Millam, M. Klene, J. E. Knox, J. B. Cross, V. Bakken, C. Adamo, J. Jaramillo, R. Gomperts, R. E. Stratmann, O. Yazyev, A. J. Austin, R. Cammi, C. Pomelli, J. W. Ochterski, R. L. Martin, K. Morokuma, V. G. Zakrzewski, G. A. Voth, P. Salvador, J. J. Dannenberg, S. Dapprich, A. D. Daniels, O. Farkas, J. B. Foresman, J. V. Ortiz, J. Cioslowski, and D. J. Fox, *Gaussian 16. Revision C.01*, Gaussian, Inc, Wallingford CT, **2019**.

[9] C. M. Cardona, W. Li, A. E. Kaifer, D. Stockdale, G. C. Bazan, *Adv. Mater.* **2011**, *23*, 2367.

[10] T. Nishinaga (Ed.) *Organic Redox Systems. Synthesis, Properties, and Applications*, John Wiley & Sons, Inc., Hoboken, New Jersey, **2016**.
